# Progressive Rotavirus Infection Downregulates Redox-Sensitive Transcription Factor Nrf2 and Nrf2-Driven Transcription Units

**DOI:** 10.1155/2020/7289120

**Published:** 2020-04-04

**Authors:** Upayan Patra, Urbi Mukhopadhyay, Arpita Mukherjee, Rakesh Sarkar, Mamta Chawla-Sarkar

**Affiliations:** Division of Virology, National Institute of Cholera and Enteric Diseases, P-33, C.I.T. Road Scheme-XM, Beliaghata, Kolkata 700010, India

## Abstract

Eukaryotic cells adopt highly tuned stress response physiology under threats of exogenous stressors including viruses to maintain cellular homeostasis. Not surprisingly, avoidance of cellular stress response pathways is an essential facet of virus-induced obligatory host reprogramming to invoke a cellular environment conducive to viral perpetuation. Adaptive cellular responses to oxidative and electrophilic stress are usually taken care of by an antioxidant defense system, core to which lies the redox-responsive transcription factor nuclear factor erythroid 2-related factor 2 (Nrf2) and Nrf2-driven transcriptional cascade. Deregulation of host redox balance and redox stress-sensitive Nrf2 antioxidant defense have been reported for many viruses. In the current study, we aimed to study the modulation of the Nrf2-based host cellular redox defense system in response to Rotavirus (RV) infection *in vitro*. Interestingly, we found that Nrf2 protein levels decline sharply with progression of RV infection beyond an initial upsurge. Moreover, Nrf2 decrease as a whole was found to be accompanied by active nuclear vacuity of Nrf2, resulting in lowered expression of stress-responsive Nrf2 target genes heme oxygenase-1 (HO-1), NAD(P)H quinone dehydrogenase 1, and superoxide dismutase 1 both in the presence and absence of Nrf2-driven transcriptional inducers. Initial induction of Nrf2 concurred with RV-induced early burst of oxidative stress and therefore was sensitive to treatments with antioxidants. Reduction of Nrf2 levels beyond initial hours, however, was found to be independent of the cellular redox status. Furthermore, increasing the half-life of Nrf2 through inhibition of the Kelch-like erythroid cell-derived protein with CNC homology- (ECH-) associated protein 1/Cullin3-RING Box1-based canonical Nrf2 turnover pathway could not restore Nrf2 levels post RV-SA11 infection. Depletion of the Nrf2/HO-1 axis was subsequently found to be sensitive to proteasome inhibition with concurrent observation of increased K48-linked ubiquitination associated with Nrf2. Together, the present study describes robust downregulation of Nrf2-dependent cellular redox defense beyond initial hours of RV infection, justifying our previous observation of potent antirotaviral implications of Nrf2 agonists.

## 1. Introduction

In response to stress insults, eukaryotic cells are endowed with the adaptability to conform over normal physiology to stress response physiology—a heightened survival response to combat stress and to regain cellular homeostasis thereafter. Core to negotiating with cellular redox stress has been an antioxidant defense system orchestrated by the nuclear factor erythroid 2-related factor 2 (Nrf2), a transcription factor belonging to the basic leucine zipper family, and Nrf2-driven transactivation of a series of stress-responsive genes. Under unstressed conditions, Nrf2 is turned over very rapidly by its interaction with Kelch-like erythroid cell-derived protein with CNC homology- (ECH-) associated protein 1 (Keap1), a cysteine-rich adaptor of the Cullin3-RING Box1 (Cul3-Rbx1) E3 ubiquitin ligase complex, which renders Nrf2 a subject of K48-linked ubiquitination and prompt proteasomal degradation. Upon oxidative challenge, conformational switch of Keap1 as a result of covalent modification of stress-responsive Keap1 cysteine subsets renders the co-“locked” Nrf2 insensitive to degradation by the ubiquitin-proteasome pathway. Nascent Nrf2 molecules, being freed of Keap1 repression, translocate to the nucleus and exert their transactivation potency by binding to the Antioxidant Response Element (ARE), the canonical cis-acting enhancer sequence (TCAG/CXXXGC) in the promoter region of Nrf2-regulated cytoprotective genes [1–7]. Owing to extreme sensitivity of cellular redox homeostasis to the minutest of perturbations, both exogenous (pathogen infection, radiation, electrophilic, and chemical stressors) and endogenous (energy deficiency and metabolic reprogramming), and interconnectivity of the Nrf2-dependent cytoprotective defense cascade with other cellular stress response pathways (heat shock response, unfolded protein response, translational arrest, autophagic/apoptotic demise, and nutrient deprivation), it is not surprising that inducibility of Nrf2 transactivation has multiple layers of regulations at transcriptional, translational, and posttranslational levels [2]. Growing body of evidence is accumulating in favour of dispensability of the Cul3-Rbx1 E3 ubiquitin ligase complex for Nrf2 suppression either in a Keap1-dependent way or even independent of Nrf2-Keap1 interaction under selective pathophysiological conditions [8]. Moreover, the status of residue-selective Nrf2 phosphorylation has been reported to have regulatory impacts on Nrf2. A number of cellular kinases (p38 Mitogen-Activated Protein Kinase (p38MAPK), c-Jun N-Terminal Kinase (JNK), Extracellular Signal-Regulated Kinase (Erk1/2), Casein Kinase II (CKII), Protein Kinase C (PKC), Phosphoinositide 3-Kinase (PI3K), Protein Kinase R-Like Endoplasmic Reticulum Kinase (PERK), and AMP-Activated Protein Kinase (AMPK)) have been documented to exert positive influence on stabilization, nuclear translocation, and transactivation potency of Nrf2 with or without redox stimuli. In contrast, a few others (Glycogen Synthase Kinase 3*β* (GSK3*β*) and Fyn tyrosine kinase) have been shown to trigger nuclear expulsion and proteasomal destruction of Nrf2 [2–4, 8]. To add complexity, translational control of Nrf2 includes augmented decoding of its open reading frame from selected internal ribosome entry sites (IRES), enabling induction of the Nrf2-based cellular defense cascade to function even under acute translational stress [9–12].

Not surprisingly, progressive viral infection essentially entails evasion of the cellular stress response menace either by antagonizing it or by usurping it noncanonically for self-perpetuation. The intricacy of host stress response biology during infection with Rotavirus (RV), an infantile diarrheagenic virus of the *Reoviridae* family, reiterates the same-viral countermeasures to outwit host defense measures. Transcriptionally competent rotaviral double-layered particles, generated by being peeled off from invading nonenveloped, triple-layered virions, potentiate production of copious positive single-stranded RNAs (+ssRNAs) from 11 segments of the double-stranded RNA (dsRNA) genome within enterocyte cytoplasm. RV +ssRNAs are further translated into six structural (VP1, VP2, VP3, VP4, VP6, and VP7) and six nonstructural proteins (NSP1, NSP2, NSP3, NSP4, NSP5, and NSP6) and also serve as replicative templates for reconstitution of the dsRNA genome within rotaviral inclusion bodies (viroplasms) [13–15]. Despite lacking sequential mechanistic details, evidences of rotaviral host subversive strategies are now ample [16–39]. Though the role of antioxidant defense elements has been studied in the case of many viruses, crosstalk between RV infection and cellular Nrf2-dependent redox defense has remained unaddressed so far.

There are compelling evidences for the Nrf2-based antioxidant pathway to be differentially regulated during the course of RV infection. Upsurge of oxidative stress during initial hours of rotaviral infection has been cited [40, 41]. Moreover, abiding by the reports of redox-independent Nrf2 regulation under the overriding influence of cellular kinases, as observed posttreatment with many Nrf2 agonists (andrographolide, *tert*-butylhydroquinone, Hemin, apigenin, and phorbol myristate acetate) [42–45], available knowledge on the kinase activation profile in RV-infected cells suffices to hypothesize robust Nrf2 induction and transactivation. Of significance, not only do the Nrf2-inducing kinases such as p38MAPK, Erk1/2, JNK [46], PI3K [22, 47–50], and AMPK [21, 38] get activated during RV infection, but also inhibition of these kinase cascades affects rotaviral propagation, implicating immense proviral importance of these cellular kinases instead of mere bystander activation [21, 22, 46, 49, 51]. Retention of constitutive kinase activity of CKII [52] and functional inactivation of the GSK3*β* downstream of PI3K activation [22] in RV-infected cells again indicate a cellular milieu conducive to Nrf2 stabilization. Contrastingly, attenuation of the host antioxidant repertoire has been reported upon RV-induced gastroenteritis in some animal model studies [53, 54]. Supportive observations documented chemically generated an antioxidative cellular environment to exert potent antagonistic effects on RV infection both *in vitro* [55] and in the mouse model of infection [56] and also to ameliorate RV-induced diarrhea in clinical patients [57]. Our recent study on potent antirotaviral efficacy of Nrf2 agonists further corroborates the possibility of the Nrf2-dependent antioxidant defense system to have an antiviral role during RV infection [58].

In the present context, we addressed the status of the Nrf2-based cellular antioxidant defense system in response to RV infection *in vitro*. Assessing protein levels of total and nuclear Nrf2 with respect to a mock-infected control as a function of time point post RV infection revealed a bimodal regulation: an initial induction followed by gradual depletion thereafter. Subsequent investigations showed only the initial upsurge of Nrf2, but not the steady decline thereafter, to be redox-regulated. Depletion of Nrf2 beyond early hours of RV infection was also transduced to attenuation of the Nrf2 target gene expression. Suppression of Nrf2 was further found out to be independent of the canonical Nrf2 turnover pathway but sensitive to proteasomal inhibition and therefore associated with increased K48-linked ubiquitination. Cumulatively, we described RV infection to trigger significant attenuation of the cellular antioxidant defense cascade by rendering degradation of the redox-responsive master transcription factor Nrf2 via the ubiquitin-proteasome pathway.

## 2. Materials and Methods

### 2.1. Cell Culture

Human colorectal adenocarcinoma cell line HT29 (ATCC number: HTB-38™) was cultured in Dulbecco's Modified Eagle Medium (DMEM), and monkey kidney cell line MA104 (ATCC number: CRL-2378™) and another RV-permissive human colorectal adenocarcinoma cell line Caco2 were cultured in Minimal Essential Medium (MEM) supplemented with 10% (*v*/*v*) heat-inactivated Fetal Bovine Serum (FBS; Gibco™) and 1% (*v*/*v*) Antibiotic-Antimycotic (Gibco™) within humidified 5% CO_2_ incubator at 37°C.

### 2.2. Virus Infection

Cell culture-adapted RV strains SA11 (simian strain; G3P[2]), A5-13 (bovine strain; G8P[1]), and KU (human strain; G1P[8]) were used in this study. All viral strains were propagated in the MA104 cell line. Extracted viral preparations were titrated and calculated by plaque assay as described previously [22, 51, 59]. Unless otherwise mentioned, cells were infected at a multiplicity of infection (moi) 3 in all experiments as detailed previously [22, 51]. The time of virus addition was considered as 0 hour post infection (0 hpi) for all experiments described throughout the manuscript. Uninfected cells were treated exactly like infected cells with the exception of adding acetylated trypsin-treated serum-free medium instead of adsorbing RV (designated as mock infected).

### 2.3. UV-Inactivation of Virus

For preparing UV-inactivated RV, SA11 was preincubated with 40 *μ*g/ml psoralen AMT (from 1 mg/ml stock solution in 50%ethanol + 50%water) for 15 minutes and subsequently irradiated with a long-wave UV light of 365 nm wavelength for 2 hours under ice-cold condition [60].

### 2.4. Reagents and Antibodies

All reagents used in this study are listed in Table 1 and reconstituted according to manufacturers' instructions. Final concentration and time of addition of each of the reagents are detailed either in Results or in the respective figure legends. Of note, working concentrations of the chemical reagents used were well below their respective cytotoxic concentrations. Polyclonal and monoclonal antibodies used for this study are listed in Table 2 and were used according to the manufacturers' recommended dilutions. Antiserum against RV-SA11 nonstructural protein 3 (NSP3) was raised in rabbits using purified full-length RV protein expressed in a bacterial expression system according to standard protocols at the Department of Virology and Parasitology, Fujita Health University School of Medicine, Aichi, Japan.

### 2.5. Subcellular Fractionation of Nucleus and Cytosol

Nuclear fractions were isolated by NE-PER™ Nuclear and Cytoplasmic Extraction Reagents (78833; Thermo Scientific™) by following the manufacturer's instructions.

### 2.6. Transfection of siRNA

Transfection of siKeap1 (QIAGEN), siRbx1 (QIAGEN), and scrambled siRNA was carried out in MA104 cells with siPORT-NeoFX (Ambion) according to the manufacturer's instructions.

### 2.7. Western Blot

Cells were washed with prechilled phosphate-buffered saline (PBS) and lysed in a radioimmunoprecipitation assay (RIPA) buffer [35] under ice-cold condition. Protein concentration was quantitated by a Pierce™ BCA Protein Assay Kit (Thermo Scientific™). Whole cell lysates, nuclear fractions, and immunoprecipitates were mixed with a protein sample buffer [35] and boiled for 10 minutes. Samples were subsequently subjected to sodium dodecyl sulphate-polyacrylamide gel electrophoresis (SDS-PAGE), transferred onto a Polyvinylidene Fluoride (PVDF) membrane, and probed with specific antibodies as described previously [22]. Primary antibodies were detected with horseradish peroxidase- (HRP-) conjugated secondary antibody (Thermo Scientific™) and chemiluminescent substrate (Millipore and Bio-Rad) within ChemiDoc Imaging System (Bio-Rad) or onto BioMax Film (Kodak). The immunoblots shown are representatives of at least three independent experiments. Band intensities were measured using Image Lab software v5.2.1 and/or ImageJ, normalized to loading control and represented as relative fold changes (with respect to the first lane unless otherwise mentioned) in bar graphs (mean ± standard deviation; *n* ≥ 3). GAPDH and Histone H3 (for nuclear fractions) were used as internal loading controls. VP6 was used as a marker for RV infection. Mean percentage reduction of proteins (colour coded) in response to RV-SA11 infection between different groups is represented over the bars.

### 2.8. Immunofluorescence

MA104 cells, grown in four-welled chambered slides (BD Pharmingen) and treated and/or infected as indicated in Results were fixed with paraformaldehyde (4% (wt/vol) in PBS) for 10 minutes at room temperature and further processed as described previously [26]. After overnight incubation with primary antibodies (anti-Nrf2, anti-HO-1, and anti-VP6) at 4°C, slides were washed and further treated with rhodamine-conjugated anti-mouse (for VP6) and DyLight488-conjugated anti-rabbit antibodies (for Nrf2 and HO-1) (Jackson Laboratories, Inc., West Grove, PA) for 2 hours in the dark in a humidified 37°C incubator. Cell nuclei were finally stained with VECTASHIELD-DAPI (mounting medium) (Vector Laboratories, Burlingame, CA) and examined under a Zeiss Axioplan microscope (63x oil immersion). A designated (yellow box) area of each panel in an Nrf2-stained column was enlarged, brightness-adjusted, and represented as a separate column. The white arrow in each of the enlarged panels indicates Nrf2 fluorescence in the nuclear compartment. For corrected total cell fluorescence (CTCF) measurement, at least 10 cells from 5 different fields from 2 biological replicates were selected and fluorescence was quantified with ImageJ. For the purpose of normalization, background areas with no fluorescence were selected. CTCF was calculated using the formula CTCF =  Integrated Density − (Area of Selected Cell × Mean Fluorescence of Background Readings) [61]. Quotient of nuclear hollowing (NH_Q_) was analyzed using the following formula: NH_Q(treatment)_ = (Nuclear CTCF_(control)_/Total Cell CTCF_(control)_)/(Nuclear CTCF_(treatment)_/Total Cell CTCF_(treatment)_).

### 2.9. Plasmids and Transfection

Full-length RV-NSP1 cloned in a pcDNA6B vector was used for ectopic expression of RV-NSP1. DN-Cul3 (a gift from Wade Harper; Addgene plasmid #15820) [62] was procured from Addgene. All plasmids were transfected in MA104 cells with Lipofectamine 2000 (Invitrogen) according to the manufacturer's instructions. Control cells were transfected with an empty vector and designated as a mock transfected control.

### 2.10. Coimmunoprecipitation

Lysates from MA104 cells treated and/or infected as described in Results were precleared on antibody-uncoupled resin. Protein concentration was measured by a Bradford assay (Sigma-Aldrich, USA) or Pierce™ BCA Protein Assay Kit (Thermo Fisher Scientific, USA). A 5-15% volume from each lysate was kept as input. Equal amounts of lysates (unless otherwise mentioned) were then subjected to coimmunoprecipitation using a Pierce Co-IP kit (#26149) according to the manufacturer's instructions. N-Ethylmaleimide (NEM) (10 mM) was added in lysis buffer as a deubiquitinase inhibitor to assess Nrf2 ubiquitination.

### 2.11. Quantitative Real-Time (qRT) PCR

Total cellular RNA was extracted by TRIzol reagent (Invitrogen) according to the manufacturer's instructions. cDNA was prepared from 100 ng of RNA using a SuperScript II Reverse Transcriptase (Invitrogen) with a random hexamer by incubating at 42°C for 1 hour. Real-time PCR reactions [35] were performed in triplicate using SYBR Green (Applied Biosystems) in StepOnePlus (Applied Biosystems) with primers listed in Table 3. Relative gene expressions were normalized to *gapdh* using the formula 2^-*ΔΔ*CT^ (ΔΔCT = ΔCT_Sample_ − ΔCT_Untreated control_; CT is the threshold cycle).

### 2.12. Knockdown of NSP3 Expression by NSP3 shRNA

Short hairpin sequences targeting NSP3 (forward primer—5′-CCGGAACAGATGGCCGTCTCAATTACTCGAGTAATTGAGACGGCCATCTGTTTTTTTG-3′; reverse primer—5′-AATTCAAAAAAACAGATGGCCGTCTCAATTACTCGAGTAATTGAGACGGCCATCTGT-3′) were generated with the siRNA Selection Program hosted by the Whitehead Institute for Biomedical Research and inserted into a PLKO.1-TRC cloning vector (Addgene plasmid #10878) [63]. MA104 cells were transfected with NSP3 shRNA using Lipofectamine 2000; knockdown efficiency of NSP3 was assessed by immunoblotting from cells expressing NSP3 shRNA using an anti-NSP3 antibody.

### 2.13. Evaluation of Oxidative Stress

For reactive oxygen species (ROS) detection using a microscopic setup, MA104 cells were seeded in glass-bottomed dishes. After indicated treatments, cell medium was replaced with PBS containing 2′,7′-dichlorofluorescin diacetate (DCFDA) (20 *μ*M) for 30 minutes at 37°C. After final washing with PBS, DCF fluorescence from live cells was excited at a 480 ± 15 nm bandpass filter and detected with a 520 nm longpass filter using the setup of a Zeiss Axioplan microscope [64].

For ROS detection using spectrofluorometry, standard protocols of a cellular ROS detection assay kit (Abcam) were followed. Briefly, MA104 cells were treated and/or infected as indicated in Results. Cells were finally washed in 1x buffer, stained with DCFDA (20 *μ*M) for 30 minutes at 37°C; fluorescence intensity was measured at Ex/Em: 485/535 in a Varioskan™ Flash Multimode Reader (Thermo Scientific).

### 2.14. Cell Viability Assay

To check cytotoxicity of chemicals in MA104 cells, cell viability assays were conducted by MTT assay. Briefly, 10 *μ*L of MTT solution (5 mg/mL in PBS) was added to the sample-treated cells and incubated at 37°C for 4 hours. The formazan complex was dissolved in 200 *μ*L MTT solvent (4 mM HCl, 0.1% Nonidet P-40 in isopropanol). Optical density (OD) was finally measured at 570 nm. Percentage of cell viability was calculated by the formula (OD_Sample_ − OD_Blank_) × 100/(OD_Control_ − OD_Blank_).

### 2.15. Statistical Analyses

Mean ± standard deviation (S.D.) or mean ± standard error of the mean (SEM) from at least three experimental replicates (*n* ≥ 3) was considered for analyses. Statistical significance of data is marked and indicated as follows: “*ns*” stands for nonsignificant; “∗,” “#,” and “$” stand for *p* < 0.05; “∗∗,” “##,” and “$$” stand for *p* < 0.01; and “∗∗∗,” “###,” and “$$$” stand for *p* < 0.001. Comparison groups for densitometric analysis, CTCF quantitation, and qRT-PCR analysis have been described in the figure legend. *p* value of <0.05 was considered to be statistically significant for all experiments. Results were compared among different groups by either one-way ANOVA analysis followed by Tukey's post hoc test (for multiple comparisons) or independent sample *t* test (between two groups) using GraphPad Prism (Version 5).

## 3. Results

### 3.1. RV Infection Triggers Gradual Decline of Nrf2 Protein Levels beyond an Initial Upsurge

**Figure 1:**
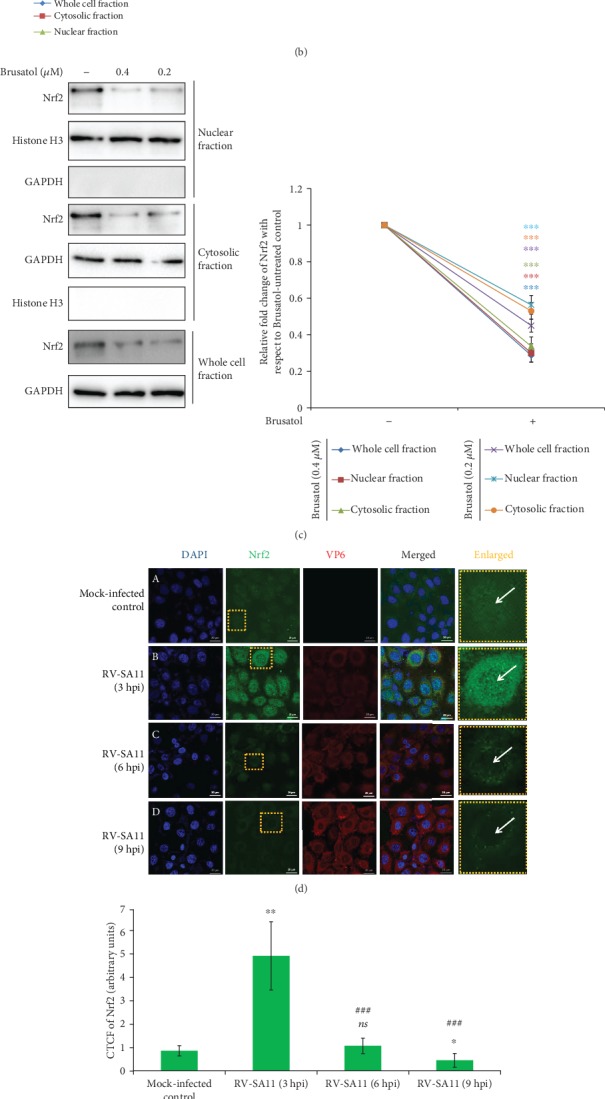
RV infection triggers gradual decline of Nrf2 protein levels beyond an initial upsurge. (a) MA104 cells were infected with either RV-SA11, UV-inactivated RV-SA11, or kept mock infected for indicated time points. Whole cell extracts were subsequently prepared and subjected to SDS-PAGE followed by immunoblotting to assess protein levels of Nrf2. Absence of viral protein (VP6 and NSP3) expression in UV-treated RV-SA11 confirmed functional inactivation of the virus. Relative fold change of Nrf2 is represented; the symbols “*ns*” and “∗” represent comparisons with respect to the first lane; “#” represents comparison with respect to the RV-SA11 group infected for 3 hours. (b) Nrf2 protein levels were assessed from whole cellular extracts as well as purified nuclear isolates and cytosolic fractions of RV-SA11/UV-irradiated RV-SA11-infected MA104 cells by immunoblot analysis. Relative fold change of Nrf2 is represented; the symbols “*ns*” and “∗” represent comparisons with respect to the first lane; “#” represents comparison with respect to the RV-SA11 group infected for 3 hours. (c) Whole cell, purified nuclear, and cytosolic extracts of MA104 cells treated with an indicated concentration of Brusatol for 3 hours were resolved on SDS-PAGE, and Nrf2 protein levels were checked by immunoblotting. Relative fold change of Nrf2 is represented; “∗” represents comparison with respect to a Brusatol-untreated (vehicle treated) group. (d) Mock-infected/RV-SA11-infected and (g) DMSO- (vehicle)/Brusatol- (0.4 *μ*M) treated (3 hours exposure) MA104 cells were fixed at indicated time point post infection/treatment and were further processed for confocal microscopy; scale bar, 20 *μ*M. (e, h) Quantification of background-normalized Nrf2 fluorescence in (e) infected and (h) Brusatol-treated set is represented as CTCF. “*ns*” and “∗” represent comparison with respect to the first lane; “#” represents comparison with respect to the RV-SA11 group infected for 3 hours. (f, i) Quotient of nuclear hollowing (NH_Q_) of Nrf2 in (f) RV-SA11-infected cells at 3, 6, and 9 hours post infection and (i) Brusatol-treated cells after 3 hours of exposure was calculated.

To address the status of redox stress response in the context of RV infection *in vitro*, we initially assessed steady-state protein levels of Nrf2, the master transcription factor orchestrating the antioxidant defense cascade, in MA104 cells infected with RV-SA11 for different time points (3, 6, 9, and 12 hours post infection). Two separate groups of cells were kept for comparison: one set mock infected and the other infected with UV-inactivated RV-SA11 (deficient in gene expression and genome replication). Interestingly, unlike in mock-infected cells with no apparent change, steady-state Nrf2 protein levels were found to get induced at 3 hours post RV-SA11 infection followed by gradual decline as infection progressed (Figure 1(a)). In response to infection with UV-inactivated RV-SA11, steady-state Nrf2 protein level kinetics remained unchanged over time (Figure 1(a)). Nrf2 protein levels in purified nuclear fractions of RV-SA11-infected cells also reduced beyond 3 hours post RV-SA11 infection (Figure 1(b)). At 3 hpi, an increase in the nuclear translocation of Nrf2 was observed (Figure 1(b)). Moreover, the degree of induction of nuclear Nrf2 slightly surpassed that of total Nrf2 in whole cellular extracts (Figure 1(b)). Decline of nuclear Nrf2 beyond 3 hours of infection, however, followed an accelerated kinetics compared to that of total Nrf2 (Figure 1(b)). Notably, the pattern of cytosolic Nrf2 as a function of a time point post RV-SA11 infection also followed the same trend as that of nuclear Nrf2, albeit less sharply (Figure 1(b)). In agreement with the previous result obtained from whole cell extracts, no change was found in time kinetics of nuclear and cytosolic Nrf2 during infection with UV-inactivated RV-SA11 (Figure 1(b)). As reported previously [65, 66], an exposure of MA104 cells to Brusatol for 3 hours also resulted in reduced Nrf2 protein levels in whole cellular extracts as well as in purified nuclear extracts (Figure 1(c)), substantiating authenticity of our results. Interestingly, the extent of nuclear Nrf2 depletion was found to be similar to that of total as well as cytosolic Nrf2 decline in Brusatol-treated cells (Figure 1(c)). Moreover, unlike in the case of RV-SA11 infection, time kinetics of nuclear Nrf2 mimicked that of total Nrf2 under condition of Brusatol treatment (data not shown). These results suggest that RV infection might trigger nuclear vacuity of Nrf2. An identical trend of steady attenuation of total Nrf2 protein levels beyond initial induction was also observed in response to RV-SA11 infection in RV-permissive human intestinal epithelial cell lines HT29 (Supplementary [Supplementary-material supplementary-material-1]) and Caco2 (Supplementary [Supplementary-material supplementary-material-1]). Moreover, much alike to simian RV strain SA11, infection with human RV strain KU and bovine RV strain A5-13 was found to trigger a matched response of Nrf2 protein level kinetics in the HT29 and MA104 cell lines, respectively (Supplementary [Supplementary-material supplementary-material-1], [Supplementary-material supplementary-material-1]). Confocal microscopy and subsequent quantification of corrected total cell fluorescence (CTCF) also revealed robust induction of Nrf2 at 3 hpi followed by gradual decline (at 6 and 9 hpi) with progression of infection (Figures 1(d) and 1(e)). Interestingly, induced Nrf2 at 3 hpi was not restricted exclusively to a nuclear compartment but was found to be distributed throughout the cell (Figure 1(d), B). Beyond 3 hpi, however, along with significant decrease in Nrf2-specific fluorescence, the nuclear compartment was particularly observed to be bereft of Nrf2—a phenomenon we termed as “nuclear hollowing” (Figure 1(d), C and D). To further quantify the extent of nuclear hollowing of Nrf2 in response to RV-SA11 infection over and above the decline in total cell Nrf2 CTCF, we calculated the “quotient of nuclear hollowing” (NH_Q_) (described in Materials and Methods). A value of NH_Q_ more than 1 for a particular treatment signifies the corresponding fold of nuclear hollowing in response to that particular treatment with respect to the control. Nuclear enrichment (a decrease of nuclear hollowing), on the other hand, shows a value of NH_Q_ between 0 and 1 on this scale; the more the nuclear aggregation (and the lesser the nuclear hollowing), the closer the NH_Q_ value is to 0. We found NH_Q_ to dip to 0.71 at 3 hpi with respect to mock-infected controls, suggesting nuclear enrichment of Nrf2 over and above the induction of total Nrf2 fluorescence at the initial time point of RV-SA11 infection (Figure 1(f)). Interestingly, NH_Q_ values were observed to increase with progression of infection (1.44 and 6.31 at 6 and 9 hpi, respectively) (Figure 1(f)), justifying the phenomenon of nuclear hollowing. Significant reduction of Nrf2 CTCF upon exposure to Brusatol (Figures 1(g) and 1(h)), however, was not found to be accompanied by nuclear hollowing (Figure 1(i)). These results, in agreement with the immunoblot data, suggest that RV infection triggers nuclear vacuity of Nrf2 along with total cell Nrf2 diminishment. To further address whether the protein level kinetics of Nrf2 in response to RV infection resulted from cellular antiviral signaling, we checked Nrf2 protein levels in MA104 cells in a time point-dependent manner following transfection with Poly(I:C), a double-stranded RNA analogue. Interestingly, administration of Poly(I:C) triggered induction of Nrf2 protein levels as a function of time post transfection (Supplementary [Supplementary-material supplementary-material-1]), nullifying the possibility of general antiviral signaling to be responsible for Nrf2 protein level attenuation observed beyond 3 hours post RV infection.

**Figure 2:**
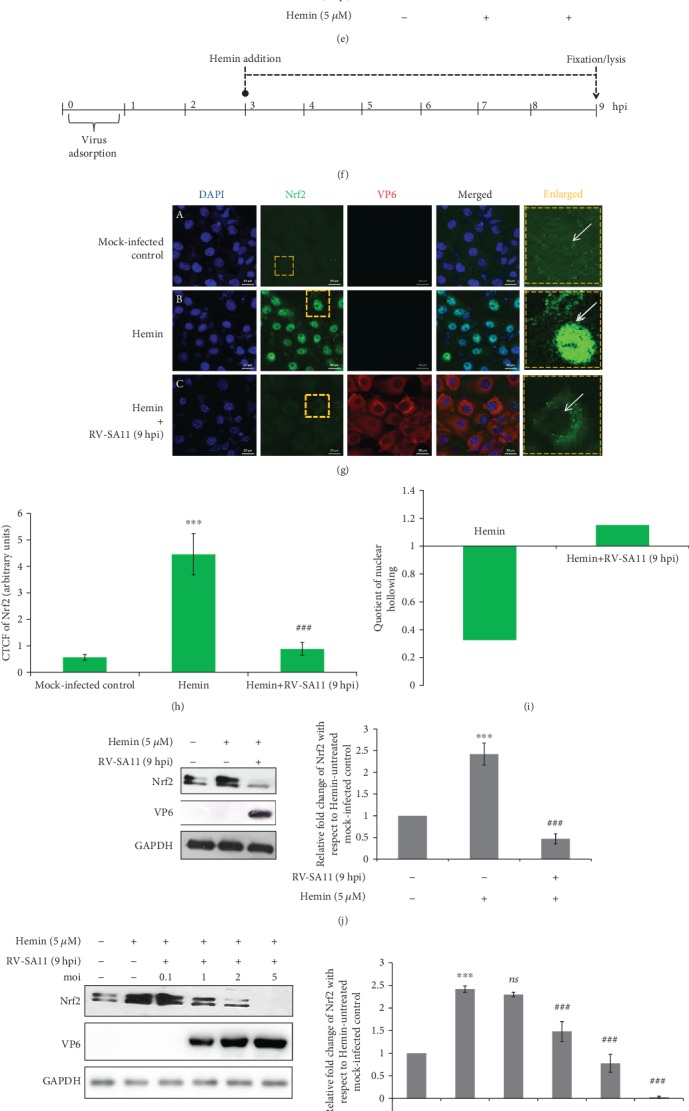
RV infection depletes Hemin-induced Nrf2 levels beyond the early hours of infection. (a, f) Time scale of Hemin addition and cell extraction/fixation with reference to RV-SA11 infection time point is schematically represented. (b–e) MA104 cells were treated/infected as shown schematically in (a) and, subsequently, either processed for (b) confocal microscopy (scale bar, 20 *μ*M) followed by (c) CTCF quantification or subjected to (e) SDS-PAGE/immunoblotting to assess Nrf2. (g–j) MA104 cells were treated/infected as shown schematically in (f) and subsequently either (g) processed for confocal microscopy (scale bar, 20 *μ*M) followed by (h) CTCF quantification or (j) subjected to SDS-PAGE/immunoblotting to assess Nrf2. (e, j) Densitometric analyses and (c, h) CTCF quantification of Nrf2 are represented; “∗” and “#” represent comparisons with respect to vehicle-treated mock-infected and Hemin- (5 *μ*M) treated mock-infected groups, respectively. (d, i) Quotient of nuclear hollowing (NH_Q_) of Nrf2 is shown in the bar graph. (k) Nrf2 protein levels were assessed by SDS-PAGE/immunoblotting in Hemin- (5 *μ*M; added at 3 hpi) treated MA104 cells infected with RV-SA11 at gradually increasing moi (0.1, 1, 2, and 5) for 9 hours. Relative fold change of Nrf2 is represented; “∗” represents comparison with respect to vehicle-treated mock-infected control; “*ns*” and “#” represent comparisons with respect to a Hemin- (5 *μ*M) treated mock-infected group. (l) MA104 cells treated with Hemin (5 *μ*M) for 3 hours were further cotreated with an indicated concentration of Brusatol for an additional 3 hours. Nrf2 protein levels were finally assessed from whole cell extracts by SDS-PAGE and immunoblot analyses. Relative fold change of Nrf2 is represented; “∗” and “#” represent comparisons with respect to vehicle-treated control and Hemin- (5 *μ*M) treated Brusatol-untreated groups, respectively. (m) Hemin-treated MA104 cells (an exposure of 3 hours) were cotreated either with Brusatol (0.4 *μ*M) or DMSO (additional exposure of 3 hours) before cell fixation and preparation for confocal imaging. One set of cells, kept as Hemin-untreated DMSO-treated control, was processed simultaneously. Scale bar, 20 *μ*M. Panel A is identical to panel A of Figure 1(g). (n) Nrf2 CTCF from each panel of (m) was shown. “∗” and “#” represent comparisons with respect to vehicle-treated control and Hemin- (5 *μ*M) treated Brusatol-untreated group, respectively. (o) Quotients of nuclear hollowing (NH_Q_) of Nrf2 in Hemin+DMSO-treated and Hemin+Brusatol-treated cells are represented with respect to the Hemin-untreated DMSO-treated control.

**Figure 3:**
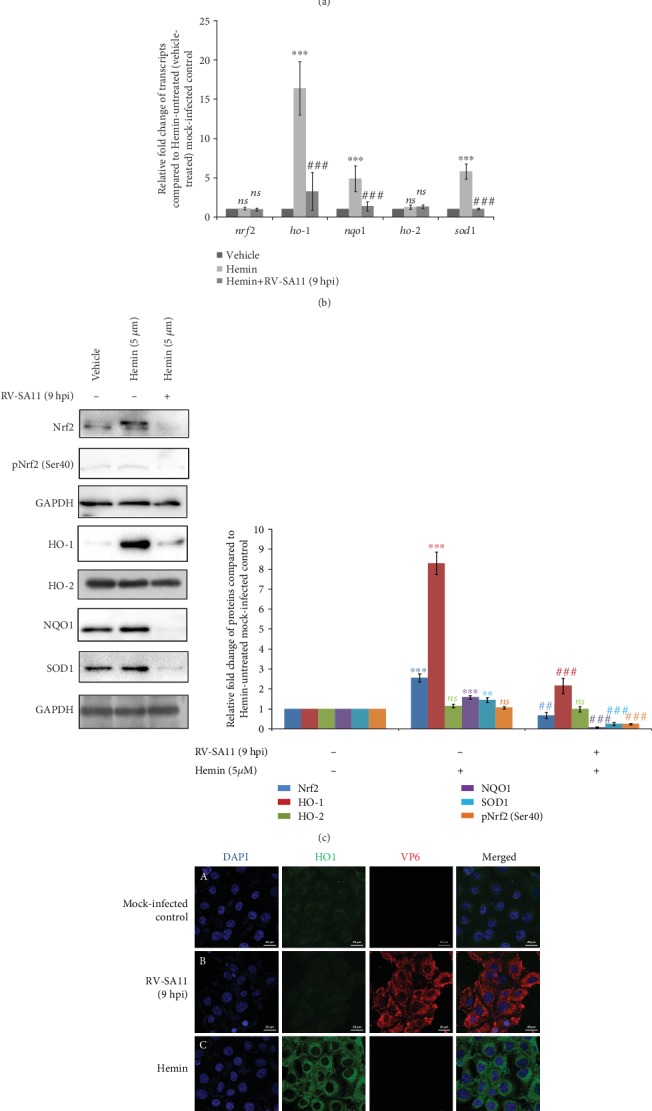
Modulation of Nrf2 during RV-SA11 infection is transduced to matched the response from Nrf2-driven transcription units. (a) Extracts from mock- and RV-SA11-infected (3, 6, 9, and 12 hours) MA104 cells were subjected to SDS-PAGE/western blotting for checking expressions of Nrf2, pNrf2 (Ser40), HO-1, HO-2, NQO1, and SOD1. Relative fold changes of proteins are represented; the symbols “*ns*” and “∗” represent comparisons with respect to the first lane; “#” represents comparison with respect to the RV-SA11 group infected for 3 hours. (b, c) Hemin-treated (5 *μ*M; added at 3 hpi) MA104 cells were either mock infected or infected with RV-SA11 (for 9 hours). One set of cells was kept as the Hemin-untreated mock-infected control. (b) mRNA and (c) protein level expressions of Nrf2, pNrf2 (Ser40), HO-1, HO-2, NQO1, and SOD1 were analyzed by (b) quantitative real-time PCR and (c) SDS-PAGE/immunoblotting. (b, c) Relative fold changes of (b) transcripts and (c) proteins are represented; the symbols “*ns*” and “∗” represent comparisons with respect to the mock-infected Hemin-untreated control; “#” represents comparison with respect to the mock-infected Hemin-treated group. (d, e) Hemin- (5 *μ*M)/vehicle- (H_2_O) treated RV-SA11-infected and mock-infected (9 hpi) cells were processed for visualization of HO-1 fluorescence by confocal microscopy. Scale bar, 20 *μ*M. (e) HO-1 CTCF from each panel of (d) was represented. “∗” and “#” represent comparisons with respect to Hemin-untreated mock-infected and Hemin-treated mock-infected groups, respectively.

Owing to low basal levels of cellular Nrf2 under unstressed condition, we further investigated whether RV infection can also decrease chemically induced Nrf2 levels beyond 3 hours post infection. For this end, we treated RV-SA11-infected cells with 5 *μ*M of Hemin either at 1 hpi (Figure 2(a)) or 3 hpi (Figure 2(f)) and assessed Nrf2 protein levels 6 hours post Hemin treatment (at 7 hpi (Figure 2(a)) and 9 hpi (Figure 2(f)), respectively). The duration of Hemin treatment was chosen to be 6 hours to ensure optimal induction of Nrf2 [43]. Confocal microscopy followed by CTCF analysis revealed induction of Nrf2 in Hemin-treated mock-infected cells compared to Hemin-untreated mock-infected control (Figures 2(b), 2(c), 2(g), and 2(h)). Interestingly, RV-SA11 infection triggered marked quenching of Hemin-induced Nrf2 both at 7 hpi (Figures 2(b) and 2(c)) and 9 hpi (Figures 2(g) and 2(h)). Similar results were obtained when the Hemin-induced Nrf2 protein expression was assessed in RV-SA11-infected cells by western blotting followed by densitometric analysis (Figures 2(e) and 2(j)). Robust nuclear hollowing of Nrf2 was distinct in Hemin-treated infected cells as heavy nuclear aggregation of Nrf2 in response to Hemin was markedly attenuated following RV infection (Figures 2(b) and 2(g)). Quantitatively, with respect to the Hemin-treated mock-infected control, RV-SA11 resulted in an increase of NH_Q_ values by 2.31- and 3.5-fold at 7 and 9 hours post infection, respectively (Figures 2(d) and 2(i)). Moreover, suppression of Hemin-driven Nrf2 protein levels at 9 hpi correlated with increasing multiplicity of infection (Figure 2(k)). Indeed, Brusatol treatment did curb accumulation of Nrf2 in response to Hemin in MA104 cells, as revealed by immunoblotting (Figure 2(l)) as well as confocal microscopy (Figures 2(m) and 2(n)). However, unlike in the case of RV infection, Brusatol could not trigger significant nuclear hollowing of Hemin-induced Nrf2 (Figure 2(m)), as evident by an only 1.3-fold increase of the NH_Q_ value in the presence of Brusatol (Figure 2(o)).

### 3.2. Attenuation of Nrf2 Protein Levels during RV Infection Results in Downregulation of Nrf2-Driven Transcription Units

To further address the functional consequences of the initial induction followed by gradual depletion of Nrf2 with progression of RV infection, levels of phospho-Nrf2 (Ser40) (pNrf2 (Ser40)) were assessed at indicated time points of infection (3, 6, 9, and 12 hpi). Phosphorylation of Nrf2 at Ser40 results in Nrf2 stabilization leading to its enhanced nuclear translocation and augmented transcriptional activation of Nrf2-dependent genes [4, 42]. The pattern of pNrf2 (Ser40) levels as a function of infection time points was found to be identical to that of basal Nrf2 (Figure 3(a)). Agreeably, time kinetics of mRNA (Supplementary [Supplementary-material supplementary-material-1]) and protein (Figure 3(a)) levels of Nrf2 transcriptional targets (HO-1, NQO1, and SOD1) post RV-SA11 infection also revealed a trend similar to that of Nrf2: time-dependent gradual decrease beyond an initial induction (except SOD1 which did not show initial induction). Relative *nrf2* mRNA in RV-SA11-infected cells, however, remained unchanged over time with respect to the mock-infected control (Supplementary [Supplementary-material supplementary-material-1]), suggesting modulation of Nrf2 during RV infection to be independent of transcriptional regulation. Moreover, concurrent to our previous observation of effects of RV infection on Hemin-induced Nrf2 protein levels, Nrf2 targets were subdued in response to RV-SA11 infection in the levels of mRNA (Figure 3(b)) as well as protein (Figure 3(c)) in Hemin-treated cells. Neither Hemin treatment nor RV infection thereafter could trigger differential expression of *nrf2* mRNA (Figure 3(b)), affirming *nrf2* transcription to be unperturbed in RV-infected cells. Notably, Heme oxygenase 2 (HO-2), a noninducible isoform of HO-1, remained unaffected during RV infection either in the absence (Supplementary [Supplementary-material supplementary-material-1]; Figure 3(a)) or presence (Figures 3(b) and 3(c)) of Hemin, emphasizing the importance of Nrf2 modulation behind regulation of Nrf2-driven target genes in RV-infected cells. Interestingly, induction of Nrf2 after 6 hours of Hemin treatment failed to accompany likewise induction of pNrf2 (Ser40) (Figure 3(c)). This observation emphasizes the point that basal level induction of Nrf2 is not always reflected in commensurate pNrf2 (Ser40) induction, indicating the latter to be a cooccurring event with the former one. Confocal microscopy further reiterated reduction of the basal as well as Hemin-induced HO-1 protein at 9 hours post RV-SA11 infection (Figures 3(d) and 3(e)). Depletion of HO-1 was also observed distinctly in Brusatol-treated cells (Supplementary [Supplementary-material supplementary-material-1]). Cumulatively, these results suggest initial upsurge followed by gradual attenuation of the Nrf2/HO-1 axis upon RV-SA11 infection.

### 3.3. Initial Induction of Nrf2 Is Dependent on RV-Induced Early Burst of Oxidative Stress and PKC

**Figure 4:**
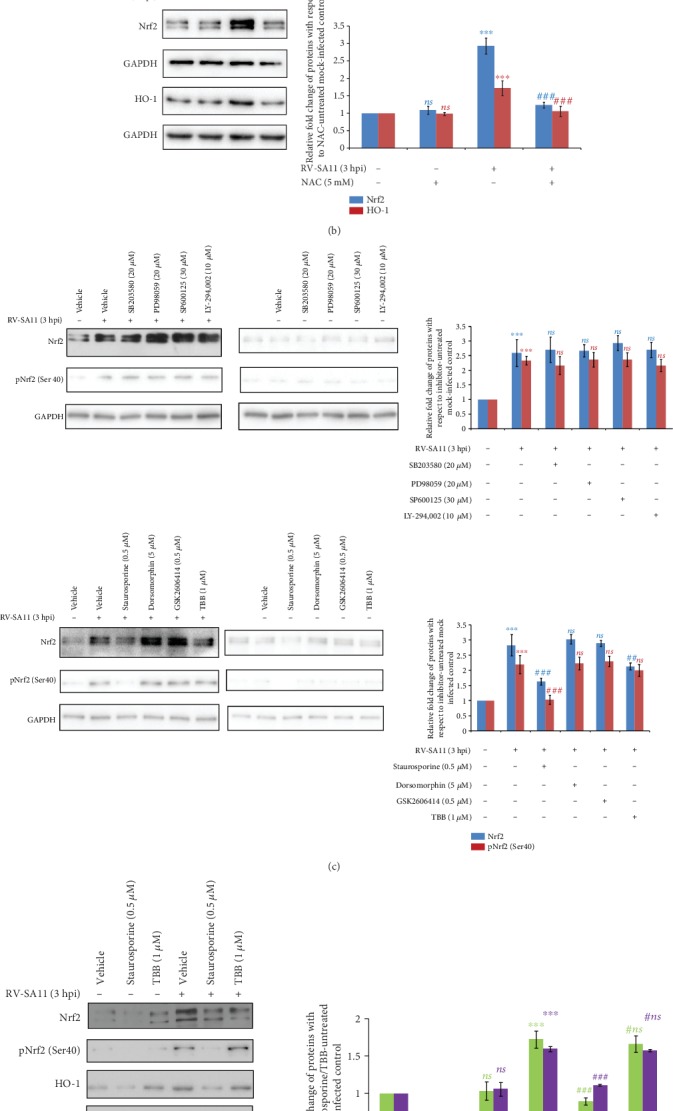
Initial induction of Nrf2 is dependent on RV-induced early burst of oxidative stress and PKC. (a) MA104 cells mock infected or infected with RV-SA11 for 3 hours were further subjected to DCFDA-based confocal imaging. A separate group of cells was treated with a well-characterized oxidative stressor sodium arsenite (NaAsO_2_; 50 *μ*M) for 2 hours before assessment of ROS induction by DCFDA-based confocal microscopy. (b) MA104 cells were mock infected or infected with RV-SA11 for 3 hours in the presence or absence of NAC (5 mM; added during final media addition). Nrf2 and HO-1 protein levels were subsequently checked in whole cell extracts by SDS-PAGE/immunoblot analyses. Relative fold changes of Nrf2 and HO-1 are represented; “*ns*” and “∗” represent comparisons with respect to vehicle-treated mock-infected control; “#” represents comparison with respect to the vehicle-treated RV-SA11-infected (3 hpi) group. (c) Nrf2 and pNrf2 (Ser40) levels were assessed in mock-infected and RV-SA11 infected cells (3 hpi) in the presence or absence of a series of kinase inhibitors (mentioned in Results) added at the indicated concentration at the time of final media addition (1 hpi). Relative fold changes of Nrf2 and pNrf2 (Ser40) are represented; “∗” represents comparison with respect to vehicle-treated mock-infected control; “*ns*” and “#” represent comparisons with respect to the vehicle-treated RV-SA11-infected (3 hpi) group. (d) MA104 cells were mock infected or infected with RV-SA11 for 3 hours in the presence or absence of Staurosporine (0.5 *μ*M)/TBB (1 *μ*M) (added during final media addition). Nrf2, pNrf2 (Ser40), HO-1, and NQO1 protein levels were subsequently checked in whole cell extracts by SDS-PAGE/immunoblot analyses. Relative fold changes of proteins are represented; “*ns*” and “‘∗” represent comparison with respect to the vehicle-treated mock-infected control; “#*ns*” and “#” represent comparison with respect to the vehicle-treated RV-SA11-infected (3 hpi) group.

Next, we investigated how induction of Nrf2 is triggered during early hours of RV infection. Oxidative stress has been reported to be the primary stimulus for stabilizing Nrf2 and mobilizing the Nrf2/ARE pathway [2–4]. We therefore checked whether induction of oxidative stress in response to RV infection caused the initial upsurge of Nrf2. Indeed, generation of ROS in response to actively replicating RV-SA11 was observed during the initial hours of infection by a 2′,7′-dichlorofluorescin diacetate (DCFDA) spectrofluorometric assay (Supplementary [Supplementary-material supplementary-material-1]). Induction of ROS in RV-SA11-infected MA104 cells at 3 hpi was further confirmed by an enhanced DCFDA-positive green signal in immunofluorescence images (Figure 4(a)). Sodium arsenite treatment was kept as a positive control in these DCFDA-based assays. Indeed, induction of ROS was observed upon exposure to sodium arsenite (Figure 4(a); Supplementary [Supplementary-material supplementary-material-1]). Interestingly, conditioning of cells with 5 mM of N-acetylcysteine (NAC), a well-characterized antioxidant, completely abrogated the RV-induced upsurge of Nrf2 and HO-1 protein levels at 3 hpi (Figure 4(b)). A similar observation was made when two other antioxidants pyrrolidine dithiocarbamate (PDTC) and diphenyleneiodonium (DPI) also compromised Nrf2 and HO-1 induction at the initial hours of RV-SA11 infection (Supplementary [Supplementary-material supplementary-material-1], [Supplementary-material supplementary-material-1]), indicating this phenomenon to be ROS-sensitive. Indeed, confocal imaging as well as a spectrofluorometric assay using DCFDA showed NAC treatment to restore the ROS level in RV-SA11-infected cells to that in mock-infected control (Supplementary [Supplementary-material supplementary-material-1], [Supplementary-material supplementary-material-1]), justifying ablated Nrf2 induction.

Phosphorylation of Nrf2 has been advocated in many instances to impart its stability [2–4, 8]. We therefore assessed the potential role of cellular kinases to trigger Nrf2 induction observed at 3 hpi of RV infection. For this end, RV-induced Nrf2 stimulation at 3 hpi was checked in the presence of a series of kinase inhibitors (SB203580 targeting p38MAPK, PD98059 targeting ERK1/2, SP600125 targeting JNK, LY-294,002 targeting PI3K, Staurosporine targeting PKC, Dorsomorphin targeting AMPK, GSK2606414 targeting PERK, and TBB targeting CKII). Results showed Nrf2 induction at 3 hours post RV-SA11 infection to get partially abrogated in the presence of the pan-PKC inhibitor Staurosporine and CKII inhibitor TBB (Figure 4(c)). Staurosporine treatment also reduced basal Nrf2 (Figure 4(c)). Moreover, phosphorylation of Nrf2 at the Ser40 residue was found to diminish in Staurosporine-treated cells (but not in TBB-treated cells) under both mock and RV-SA11 infection (3 hpi) scenarios (Figure 4(c)). For further confirmation, we checked the sensitivity of Nrf2 targets HO-1 and NQO1 to Staurosporine and TBB treatment in the mock- and RV-SA11-infected (3 hpi) cells. Results showed PKC inhibition (but not CKII inhibition) to reduce basal and induced levels of Nrf2 targets (HO-1 and NQO1) in mock- and RV-SA11- (3 hpi) infected cells, respectively (Figure 4(d)). Consistently, another PKC inhibitor Gö 6983 also showed reduction of Nrf2, pNrf2 (Ser40), HO-1, and NQO1 protein levels in mock-infected as well as RV-SA11- (3 hpi) infected cells (Supplementary [Supplementary-material supplementary-material-1]). Of note, effects of pretreatment with the kinase inhibitors mimicked postinfection treatment effects (data not shown). Together, the induction of the Nrf2/ARE pathway during early hours of RV infection was found to be dependent on RV-mediated oxidative burst and on PKC.

### 3.4. Depletion of Nrf2/HO-1 at Late Hours of RV Infection Is Independent of Redox Regulation and Nrf2 Posttranslational Modifications

**Figure 5:**
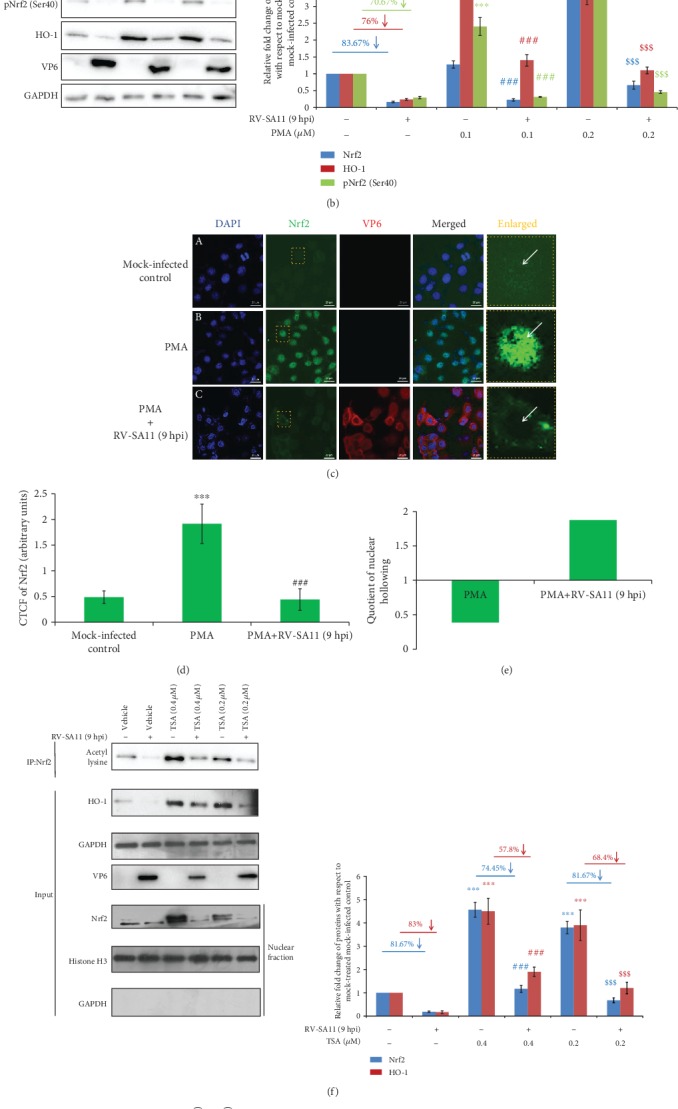
Depletion of the Nrf2/HO-1 axis beyond the early hours of RV-SA11 infection is independent of redox regulation and Nrf2 posttranslational modifications. (a) Mock-infected and RV-SA11-infected MA104 cells were treated with sodium arsenite (NaAsO_2_; 25 *μ*M and 50 *μ*M) or vehicle control (H_2_O) for 2 hours (added at 7 hpi) before cellular extract preparation at 9 hpi. Protein levels of Nrf2 and HO-1 were finally studied by SDS-PAGE/immunoblotting. Relative fold changes of proteins are represented; “∗,” “#,” and “$” represent comparisons with respect to vehicle-treated mock-infected, NaAsO_2_- (50 *μ*M) treated mock-infected, and NaAsO_2_- (25 *μ*M) treated mock-infected groups, respectively. (b–e) Mock-infected and RV-SA11-infected MA104 cells were treated with PMA (0.1 *μ*M and 0.2 *μ*M) or vehicle control (ethanol) at the time of final media addition (at 1 hpi). (b) Cellular extracts were prepared at 9 hpi for assessing protein levels of Nrf2, pNrf2 (Ser40), and HO-1 by immunoblot studies. Relative fold changes of proteins are represented; “∗,” “#,” and “$” represent comparisons with respect to vehicle-treated mock-infected, PMA- (0.1 *μ*M) treated mock-infected, and PMA- (0.2 *μ*M) treated mock-infected groups, respectively. (c) Cells fixed at 9 hpi were processed for confocal microscopy to visualize Nrf2. Scale bar, 20 *μ*M. (d) Nrf2 CTCF from each panel of (c) was shown. “∗” and “#” represent comparisons with respect to vehicle-treated mock-infected and PMA- (0.2 *μ*M) treated mock-infected groups, respectively. (e) Quotients of nuclear hollowing (NH_Q_) of Nrf2 in PMA-treated+mock-infected and PMA-treated+RV-SA11-infected cells are represented with respect to the vehicle- (ethanol) treated+mock-infected control. (f) Mock-infected and RV-SA11-infected MA104 cells were treated with TSA (0.2 and 0.4 *μ*M, respectively) or vehicle control (DMSO) (added during final media addition). Cellular lysates prepared at 9 hpi were immunoprecipitated with anti-Nrf2 antibody, and levels of acetyl lysine were checked in the immunoprecipitate by immuonblotting. Levels of HO-1 (from unfractionated input lysates) and Nrf2 (from nuclear extracts) were assessed simultaneously. Relative fold changes of proteins are represented; “∗,” “#,” and “$” represent comparisons with respect to vehicle-treated mock-infected, TSA- (0.4 *μ*M) treated mock-infected, and TSA- (0.2 *μ*M) treated mock-infected groups, respectively. (g) Mock-infected and RV-SA11-infected MA104 cells were treated with Tunicamycin (TM; 5 *μ*M and 10 *μ*M, respectively) or vehicle control (DMSO) 2 hours before cellular extract preparation at 9 hpi for assessing protein levels of Nrf2, HO-1, p-PERK, and PERK by immunoblot analyses. Relative fold changes of proteins are represented; “∗,” “#,” and “$” represent comparisons with respect to vehicle-treated mock-infected, TM- (5 *μ*M) treated mock-infected, and TM- (10 *μ*M) treated mock-infected groups, respectively.

Next, we addressed whether the reduction of Nrf2 levels beyond 3 hours of RV infection was also a response of redox stress regulation. Interestingly, reduction of the Nrf2 protein at 9 hours post RV-SA11 infection was found to be persistent even in the presence of an exogenous oxidative stressor sodium arsenite (Figure 5(a)). Dispensability of redox regulation was further confirmed when HO-1 reduction in response to RV-SA11 infection was also not rescued upon sodium arsenite treatment (Figure 5(a)). Indeed, confocal imaging with DCFDA showed sodium arsenite to induce oxidative stress during infection (Supplementary [Supplementary-material supplementary-material-1]). We further ruled out PKC to have any modulatory impact on decreased Nrf2 levels at the later phase of RV infection as treatment with the PKC inducer phorbol myristate acetate (PMA) failed to stabilize Nrf2 and HO-1 (Figure 5(b)). Moreover, consistent with the sensitivity of PMA-induced Nrf2 levels to RV-SA11 infection, PMA-induced pNrf2 (Ser40) levels also declined post (9 hours) RV-SA11 infection (Figure 5(b)). Corroborative studies on Nrf2 by confocal microscopy reiterated that strong upregulation and nuclear translocation of Nrf2 upon PMA treatment got heavily quenched at 9 hours post RV-SA11 infection (Figures 5(c) and 5(d)). We also observed a NH_Q_ value of 0.39 in PMA-treated mock-infected cells to increase to 1.88 in PMA-treated RV-SA11-infected cells (Figure 5(e)). This substantiated the occurrence of nuclear hollowing of Nrf2 beyond the early hours of RV-SA11 infection.

Acetylation-deacetylation cycles of Nrf2 have been shown to exert critical regulatory effects on nuclear stabilization and transactivation potency of Nrf2 [67, 68]. To rule out the possibility of acute Nrf2 deacetylation being the reason behind depletion of the Nrf2/HO-1 axis at late hours of RV infection, we used histone deacetylase inhibitor Trichostatin A (TSA). TSA-treated mock-infected cells showed increased fraction of acetylated Nrf2 as well as a total pool of nuclear Nrf2 (Figure 5(f)). Upon RV-SA11 infection (9 hours), however, decline in the protein levels of nuclear Nrf2 was also found to be reflected in the depletion of acetylated Nrf2 fraction (Figure 5(f)). Consistently, TSA-stimulated HO-1 levels were also lowered in RV-SA11-infected cells (Figure 5(f)), nullifying the possibility of a deregulated Nrf2 acetylation-deacetylation status to contribute to downregulation of the Nrf2/HO-1 axis during RV infection.

Previous reports have documented activation of unfolded protein response (UPR) in RV-infected cells [27, 28]. UPR has been shown to be a classical inducer of the Nrf2/ARE pathway by increasing Nrf2 in a PERK-dependent manner [69, 70]. Interestingly, even though PERK was autophosphorylated in response to Tunicamycin (TM), an ER stress inducer, in both mock-infected and RV-SA11-infected cells, TM treatment could not result in stabilization of Nrf2/HO-1 proteins during RV-SA11 infection (9 hpi) (Figure 5(g)).

### 3.5. Attenuation of Nrf2 during RV Infection Is Independent of Nrf2 Negative Regulator Keap1

**Figure 6:**
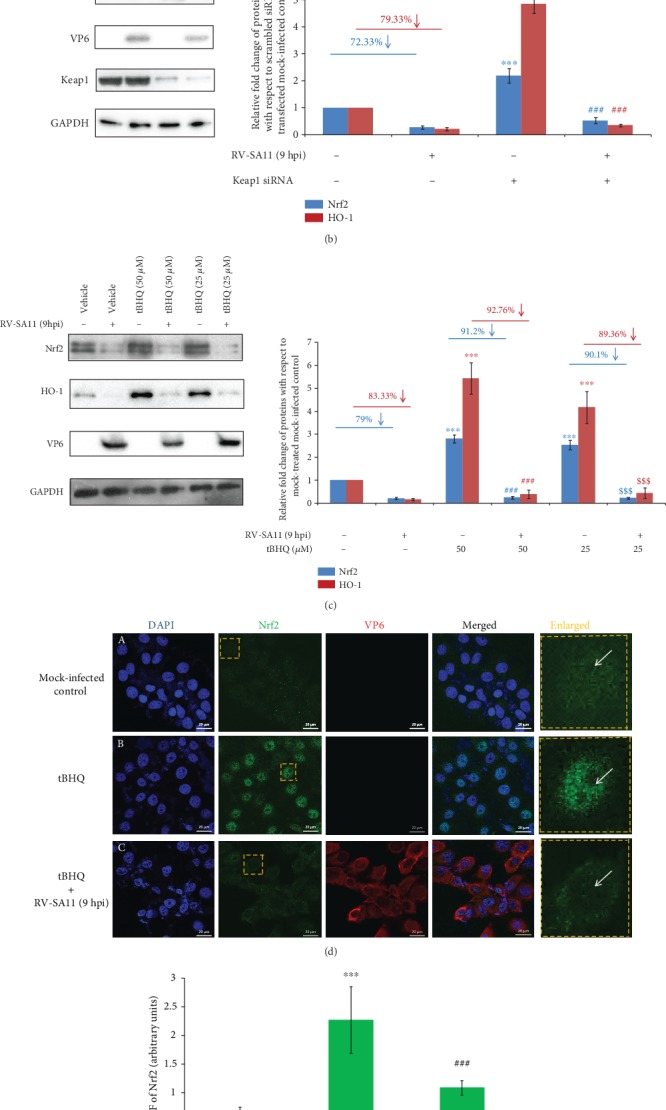
Attenuation of Nrf2/HO-1 during RV infection is independent of Nrf2 negative regulator Keap1. (a) Keap1 protein levels were checked in MA104 cells at indicated time points post RV-SA11 infection. Relative fold change of Keap1 is represented; “*ns*” and “∗” represent comparisons with respect to the mock-infected control. (b) Scrambled siRNA/Keap1 siRNA transfected MA104 cells (kept for 36 hours) were either mock infected or infected with RV-SA11 for 9 hours before cellular extract preparation followed by SDS-PAGE/immunoblot analyses for assessing protein levels of Nrf2, HO-1, and Keap1. Relative fold changes of proteins are represented; “∗” and “#” represent comparisons with respect to scrambled siRNA transfected mock-infected and Keap1 siRNA transfected mock-infected groups, respectively. (c) Lysates from mock-infected and RV-SA11-infected (9 hpi) MA104 cells cotreated with tBHQ (25 *μ*M and 50 *μ*M; added at 1 hpi) or vehicle control (ethanol) were subjected to SDS-PAGE/immunoblotting for analyzing protein levels of Nrf2 and HO-1. Relative fold changes of proteins are represented; “∗,” “#,” and “$” represent comparisons with respect to vehicle-treated mock-infected, tBHQ- (50 *μ*M) treated mock-infected, and tBHQ- (25 *μ*M) treated mock-infected groups, respectively. (d) Mock-infected and RV-SA11-infected MA104 cells were treated with tBHQ (50 *μ*M) or vehicle control (ethanol) at the time of final media addition. Cells fixed at 9 hpi were processed for confocal microscopy to visualize Nrf2. Scale bar, 20 *μ*M. (e) Nrf2 CTCF from each panel of (d) was shown. “∗” and “#” represent comparisons with respect to vehicle-treated mock-infected and tBHQ- (50 *μ*M) treated mock-infected groups. (f) Quotients of nuclear hollowing (NH_Q_) of Nrf2 in tBHQ-treated+mock-infected and tBHQ-treated+RV-SA11-infected cells were represented with respect to the vehicle- (ethanol) treated+mock-infected control.

In unstressed cells, Nrf2 remains under the repressor activity of Keap1 and is turned over rapidly via the Cullin RING Ligase (CRL) complex [71–73]. To check whether reduced Nrf2 levels beyond 3 hours post RV infection are due to the aggravated repressor activity of Keap1, the protein level of this Nrf2 inhibitory protein was assessed in a time point-dependent manner during infection. Interestingly, the Keap1 level was found to be unchanged up to 9 hpi with a modest decrease at 12 hpi (Figure 6(a)), suggesting RV-induced Nrf2 regulation to be independent of Keap1. For further confirmation, MA104 cells were silenced for Keap1 expression by Keap1 siRNA before checking the status of Nrf2 protein levels post RV-SA11 infection (9 hpi). Indeed, downregulation of Keap1 expression in mock-infected cells caused elevation of Nrf2 (Figure 6(b)). However, we found the extent of Nrf2 depletion in response to RV infection to be similar in scrambled siRNA and siKeap1 transfected cells (Figure 6(b)). Consistently, RV-mediated decrease of HO-1 remained unperturbed in cells silenced for Keap1 expression (Figure 6(b)), suggesting downregulation of the Nrf2/HO-1 axis in response to RV infection to be independent of Keap1 repression. Brusatol, as reported previously, was also found to overwhelm elevated Nrf2 in Keap1 siRNA expressing MA104 cells [66] (Supplementary [Supplementary-material supplementary-material-1]).

To finally nullify the possibility of enhanced Keap1 repression to trigger the downregulation of the Nrf2/HO-1 axis during RV infection, we used pharmacological activators of Nrf2 which function primarily by inhibiting Keap1. One of the Keap1-dependent Nrf2 inducers, *tert*-butylhydroquinone (tBHQ), has been reported to cause increased Keap1 ubiquitination resulting in its depletion possibly via the proteasome-independent way [74]. Interestingly, in agreement with our previous observation, induced levels of both Nrf2 and HO-1 in response to the dose-dependent tBHQ treatment were found to be depleted in RV-SA11-infected cells at 9 hpi (Figure 6(c)). We also observed induction and nuclear enrichment of Nrf2 in tBHQ-treated mock-infected cells to get reversed in response to infection with RV-SA11 by confocal microscopy (Figures 6(d) and 6(e)). The quotient of nuclear hollowing increased by 3.14-fold in tBHQ-treated RV-infected cells compared to tBHQ-treated mock-infected group (Figure 6(f)). Elevated Nrf2 and HO-1 levels in the presence of both irreversible Keap1 inhibitor CDDO-Me [75, 76] and reversible Keap1 inhibitor RA-839 [58, 77] underwent severe depletion under the RV infection scenario (Supplementary [Supplementary-material supplementary-material-1], [Supplementary-material supplementary-material-1]), firmly establishing Keap1-independent downregulation of the Nrf2/HO-1 axis during RV infection. It is important to mention here that all these Nrf2 inducers were used at a concentration where they were able to stabilize Nrf2 but had minimal antiviral and cytotoxic effects.

### 3.6. Cullin3/Rbx1 Complex Is Dispensable for Downregulation of Nrf2/ARE Pathway during RV Infection

**Figure 7:**
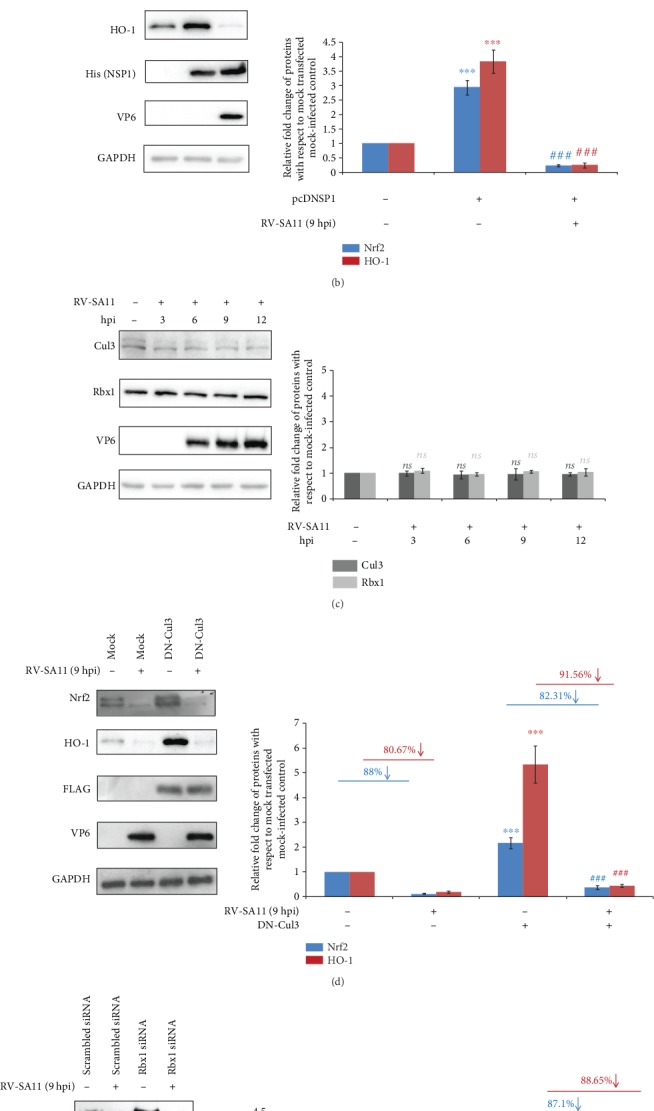
Cullin 3/Rbx1 complex is dispensable for the downregulation of the Nrf2/ARE pathway during RV infection. (a) MA104 cells were transfected with pcDNSP1 or empty vector (pcDNA6B). Levels of Nrf2, HO-1, and His (NSP1) were checked in the cellular extracts 36 hours after transfection. Relative fold changes of proteins are represented; “∗” represents comparison with respect to the mock transfected control. (b) pcDNSP1 transfected MA104 cells were infected with RV-SA11 (9 hpi) 36 hours post transfection before assessing Nrf2, HO-1, and His (NSP1) protein levels by SDS-PAGE/immunoblot analyses. Relative fold changes of proteins are represented; “∗” and “#” represent comparisons with respect to mock transfected mock-infected and pcDNSP1 transfected mock-infected groups, respectively. (c) Steady-state levels of Rbx1 and Cul3 were checked in RV-SA11-infected MA104 cells harvested at indicated time points post infection. Relative fold changes of proteins are represented; “*ns*” represents comparison with respect to the mock-infected control. (d, e) MA104 cells pretransfected with (d) DN-Cul3 and (e) Rbx1 siRNA for 36 hours were subsequently infected with RV-SA11 for 9 hours. Cellular lysates were further subjected to SDS-PAGE/immunoblot analyses and probed to check protein levels of Nrf2 and HO-1. Expressions of (d) FLAG and (e) Rbx1 were assessed to assure efficient transfection. (d, e) Relative fold changes of proteins are represented; “∗” and “#” represent comparisons with respect to mock transfected mock-infected and (d) DN-Cul3 and (e) Rbx1 siRNA transfected mock-infected groups, respectively.

RV-NSP1 has been shown to be responsible for the reduction of an array of host factors by the proteasomal [19, 39, 78] and nonproteasomal pathways [79]. Moreover, recent reports have advocated in favour of RV-NSP1 to hijack host Cullin E3 ubiquitin ligase machinery, especially the scaffolding protein Cullin3 and RING Box containing protein Rbx1 for the degradation of *β*-TrCP [80, 81]. Owing to the involvement of both Cullin3 and Rbx1 in the canonical turnover pathway of Nrf2, we speculated whether RV-NSP1 can trigger Nrf2 attenuation by the co-opted Cul3/Rbx1 complex. Surprisingly, in comparison with empty-vector transfected cells, we observed increased Nrf2 levels in the presence of ectopically expressed RV-NSP1 (Figure 7(a)). Elevated Nrf2 levels were also transduced to increased protein levels of the Nrf2 transcriptional target HO-1 under the RV-NSP1 transfected condition (Figure 7(a)). This result prompted us to investigate the status of Nrf2/HO-1 axis in RV-NSP1 transfected cells when RV infection is superimposed. Indeed, induced levels of both Nrf2 and HO-1 which were apparent in only RV-NSP1 transfected cells were found to be overridden during the infection scenario (Figure 7(b)). We further assessed protein levels of Cullin3 and Rbx1 at different time points post RV infection. Results showed no significant change in the steady-state protein levels of Cullin3 and Rbx1 (Figure 7(c)), suggesting RV-induced attenuation of the Nrf2/HO-1 axis to be independent of the Cul3/Rbx1 complex. For further assurance, we achieved loss of function of both Cullin3 and Rbx1 separately before checking levels of Nrf2 and HO-1 in response to RV-SA11 infection. Cul3 was rendered functionally inactive by overexpressing the dominant negative form of Cullin3 whereas Rbx1 expression was silenced through Rbx1 siRNA. Consistent to our previous data, loss-of-function of either Cul3 or Rbx1 did not have any effect on the RV-SA11-mediated reduction of the Nrf2 and HO-1 expressions (Figures 7(d) and 7(e)). Notably, similar to RV-infected cells, Nrf2 exhaustion in response to Brusatol treatment was also not reversed in the presence of Rbx1 siRNA (Supplementary [Supplementary-material supplementary-material-1]). Together, these results suggest that the depletion of the Nrf2/HO-1 axis beyond the initial hours of RV infection is not dependent on the canonical Nrf2 turnover pathway.

### 3.7. Downregulation of Nrf2/HO-1 Axis Is Not due to Translational Arrest or Abrogated Nuclear mRNA Export

**Figure 8:**
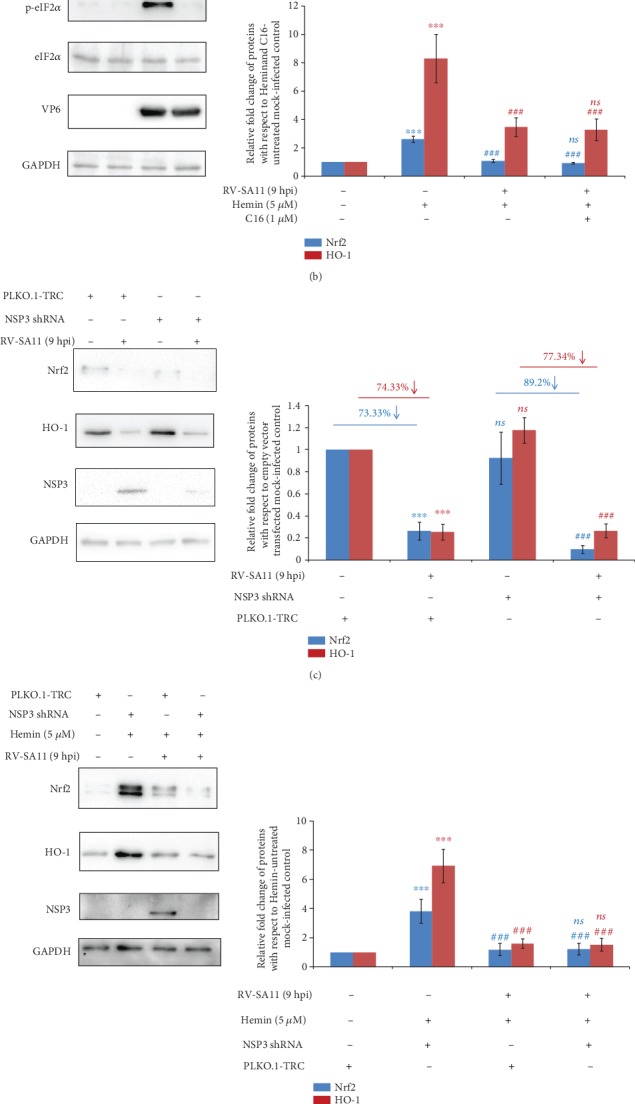
Neither translational arrest nor inhibited nuclear export of mRNA triggers downregulation of the Nrf2/HO-1 axis during RV infection. (a) Mock-infected and RV-SA11-infected MA104 cells were treated with C16 (0.5 *μ*M and 1 *μ*M) or vehicle control (DMSO) during final media addition (1 hpi). Protein levels of Nrf2, HO-1, p-eIF2*α*, and eIF2*α* were assessed in cellular extracts prepared at 9 hpi by immunoblot analyses. Relative fold changes of proteins are represented; “∗,” “#,” and “$” represent comparisons with respect to vehicle-treated mock-infected, C16- (0.5 *μ*M) treated mock-infected, and C16- (1 *μ*M) treated mock-infected groups, respectively. (b) Mock-infected and RV-SA11-infected MA104 cells were treated with Hemin (5 *μ*M; added at 3 hpi) and C16 (1 *μ*M; added at 1 hpi)/DMSO (vehicle control). Protein levels of Nrf2, HO-1, p-eIF2*α*, and eIF2*α* were finally assessed in cellular extracts prepared at 9 hpi by immunoblot analyses. Relative fold changes of proteins are represented; “∗,” “#,” and “*ns*” represent comparisons with respect to vehicle-treated mock-infected, Hemin-treated mock-infected, and Hemin-treated infected groups not treated with C16, respectively. (c) MA104 cells transfected with empty vector (PLKO.1-TRC) or NSP3 shRNA for 24 hours were further mock infected or infected with RV-SA11. Cellular extracts prepared at 9 hpi were analyzed by SDS-PAGE/western blotting to assess protein levels of Nrf2, HO-1, and RV-NSP3. Relative fold changes of proteins are represented; “*ns*” and “∗” represent comparisons with respect to the PLKO.1-TRC transfected mock-infected control; “#” represents comparison with respect to the NSP3 shRNA transfected mock-infected group. (d) Hemin-treated (5 *μ*M, added at 3 hpi) MA104 cells were either infected with RV-SA11 in the presence or absence of pretransfected NSP3 shRNA or kept mock infected. Cellular extracts prepared at 9 hpi were checked for protein levels of Nrf2, HO-1, and RV-NSP3 by SDS-PAGE/immunoblotting. Relative fold changes of proteins are represented; “∗,” “#,” and “*ns*” represent comparisons with respect to the Hemin-untreated mock-infected group transfected with PLKO.1-TRC, Hemin-treated mock-infected group, and Hemin-treated RV-SA11-infected group transfected with PLKO.1-TRC, respectively. (e) MA104 cells were treated with H_2_O_2_ (0.5 mM and 1 mM) for 2 hours. Levels of p-eIF2*α*, eIF2*α* (from whole cell extracts), Nrf2 (from whole cellular extracts as well as nuclear fractions), and PABPC1 (from nuclear fraction) were investigated by SDS-PAGE/immunoblotting. Relative fold change of Nrf2 is represented; “∗” represents comparison with respect to the H_2_O_2_-untreated control group.

Rotavirus infection has been reported to trigger severe shut-off of host cellular protein synthesis which might have further impact on RV-mediated depletion of Nrf2 and HO-1 levels. One of the key mechanisms of global translational arrest in response to RV infection is the phosphorylation of eIF2*α* by PKR resulting in inhibition of translation initiation [82]. We therefore checked whether inhibition of PKR by a chemical inhibitor C16 can block RV-mediated decrease of Nrf2 and HO-1 levels. Results showed phosphorylation of eIF2*α*, which is induced in infected cells, to get restored to the basal level upon PKR inhibition (Figure 8(a)). Interestingly, even in the absence of eIF2*α* phosphorylation in C16-treated RV-SA11-infected cells, reduction in protein levels of Nrf2 and HO-1 was evident (Figure 8(a)). Moreover, Hemin-induced Nrf2 and HO-1 levels, which were substantially reduced in RV-SA11-infected cells, were not derepressed upon PKR inhibition (Figure 8(b)). These results suggest that global translational arrest due to eIF2*α* phosphorylation was not responsible for downregulation of the Nrf2/HO-1 axis in RV-infected cells. Rotaviral NSP3 has been reported to trigger relocation of the cytoplasmic mRNA binding protein PABPC1 to the nucleus resulting in shut-off of nucleocytoplasmic shuttling of host mRNAs [83]. To further rule out the possibility of jeopardized mRNA export from the nucleus to modulate attenuation of the Nrf2/HO-1 axis post RV-SA11 infection, RV-NSP3 expression during infection was silenced by RNA interference. Depletion of both basal and Hemin-induced Nrf2/HO-1 at 9 hpi remained unperturbed even after silencing of the NSP3 expression (Figures 8(c) and 8(d)). The Nrf2/ARE pathway is one of the foremost and important types of cellular defense feedback elicited upon treatment with a variety of cellular stressors, including the ones causing global translational arrest such as oxidative stress. Interestingly, we observed strong upregulation and nuclear translocation of Nrf2 to accompany phosphorylation of eIF2*α* upon an exposure of cells to H_2_O_2_ for 2 hours (Figure 8(e)), confirming induction of the cellular redox defense system to occur even during global translational arrest. Moreover, as reported previously [84], nuclear translocation of PABPC1 was also observed in H_2_O_2_-treated cells (Figure 8(e)), emphasizing the importance of the Nrf2-based cellular defense system to reattain cellular homeostasis even under the condition of acute translational stress.

Given the vulnerability of proteins with short half-lives to suffer more from global translational arrest, we further checked whether other proteins with short half-lives were regulated similarly during RV infection. Interestingly, none of the other proteins with short half-lives such as p53, p21, c-fos, and Cyclin D1 followed the pattern of gradual reduction as infection progressed (Supplementary [Supplementary-material supplementary-material-1]). These results indicate that the inhibitory effect of RV infection on the Nrf2/HO-1 axis is specific and not a consequence of a broader effect of infection on host cellular protein synthesis.

### 3.8. Attenuation of Nrf2/HO-1 Axis Is Sensitive to Proteasome Inhibition

**Figure 9:**
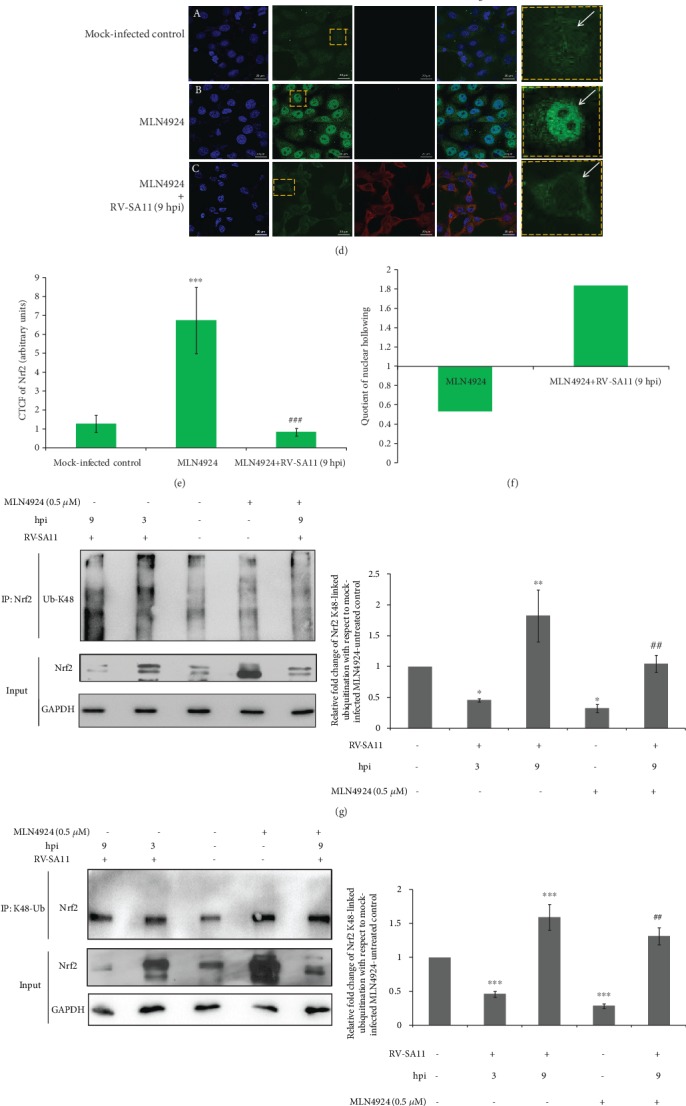
RV-mediated attenuation of Nrf2 is sensitive to proteasome inhibition and associated with increased K48-linked ubiquitination. (a, b) Mock-infected and RV-SA11-infected MA104 cells were treated with MG132 (5 *μ*M)/vehicle control (DMSO) during final media addition (1 hpi) before harvesting at (a) 6 hpi and (b) 9 hpi. Protein levels of Nrf2, HO-1, and IRF3 were subsequently analyzed from cellular extracts by SDS-PAGE/immunoblotting. Relative fold changes of proteins are represented; “∗” and “*ns*” represent comparisons with respect to vehicle-treated mock-infected and MG132- (5 *μ*M) treated mock-infected groups, respectively. (c) Lysates from mock-infected and RV-SA11-infected (9 hpi) MA104 cells cotreated with MLN4924 (0.5 *μ*M and 1 *μ*M; added at 1 hpi) or vehicle control (DMSO) were subjected to SDS-PAGE/immunoblotting for analyzing protein levels of Nrf2 and HO-1 and Cullin3. The neddylated form of Cullin3 is marked by an arrow. Relative fold changes of proteins are represented; “∗,” “#,” and “$” represent comparisons with respect to vehicle-treated mock-infected, MLN4924- (1 *μ*M) treated mock-infected, and MLN4924- (0.5 *μ*M) treated mock-infected groups, respectively. (d–f) Mock-infected and RV-SA11-infected MA104 cells were treated with MLN4924 (1 *μ*M) or vehicle control (DMSO) at the time of final media addition. Cells fixed at 9 hpi were processed for (d) confocal microscopy to visualize Nrf2. Scale bar, 20 *μ*M. (e) Nrf2 CTCF from each panel of (d) is shown. “∗” and “#” represent comparisons with respect to vehicle-treated mock-infected and MLN4924- (1 *μ*M) treated mock-infected groups, respectively. (f) Quotients of nuclear hollowing (NH_Q_) of Nrf2 in MLN4924-treated+mock-infected and MLN4924-treated+RV-SA11-infected cells were represented with respect to vehicle- (DMSO-) treated+mock-infected control. (g) Lysates from mock- and RV-SA11-infected (3 and 9 hours) MA104 cells were immunoprecipitated with anti-Nrf2 antibody. Similarly, lysates from MLN4924- (0.5 *μ*M) treated RV-SA11-infected (9 hours) and mock-infected MA104 cells were immunoprecipitated with anti-Nrf2 antibody. Immunoprecipitates were subjected to SDS-PAGE/immunoblotting with anti-K48-linked Ub antibody. The presence of Nrf2 was evaluated in input lysates. Relative fold change of K48-linked ubiquitinated Nrf2 was assessed after normalization with respective input lanes. “∗” and “#” represent comparisons with respect to MLN4924-untreated mock-infected and MLN4924-treated mock-infected groups, respectively. (h) Lysates from mock- and RV-SA11-infected (3 and 9 hours) MA104 cells were immunoprecipitated with the anti-K48-Ub antibody. Similarly, lysates from MLN4924- (0.5 *μ*M) treated RV-SA11-infected (9 hours) and mock-infected MA104 cells were immunoprecipitated with the anti-K48-Ub antibody. Immunoprecipitates were subjected to SDS-PAGE/immunoblotting with the anti-Nrf2 antibody. The presence of Nrf2 was evaluated in input lysates. Relative fold change of K48-linked ubiquitinated Nrf2 was assessed after normalization with respective input lanes. “∗” and “#” represent comparisons with respect to MLN4924-untreated mock-infected and MLN4924-treated mock-infected groups, respectively. (i, j) Lysates from MLN4924- (0.5 *μ*M) treated RV-SA11-infected (9 hours) and mock-infected MA104 cells were immunoprecipitated with (i) anti-Nrf2 or (j) anti-K48-Ub antibody. Similarly, lysates from mock- and RV-SA11-infected (3 and 9 hours) MA104 cells were immunoprecipitated with (i) anti-Nrf2 or (j) anti-K48-Ub antibody. The amount of cellular lysates which were subjected to immunoprecipitation (to assess K48-linked ubiquitinated Nrf2) was normalized on the basis of prior normalization of Nrf2 input levels such that Nrf2 levels remain the same in each input lane. Immunoprecipitates were subjected to SDS-PAGE/immunoblotting and further probed with (i) anti-K48-Ub antibody and (j) anti-Nrf2 antibodies. (k, l) Mock-infected and RV-SA11-infected MA104 cells were treated with MG132 (5 *μ*M) during final media addition (1 hpi) before harvesting at 9 hpi. Cellular lysates were immunoprecipitated with (k) anti-Nrf2 antibody or (l) anti-K48-Ub antibody. Immunoprecipitates were finally run on SDS-PAGE, transferred on to a PVDF membrane, and probed with (k) anti-K48-Ub or (l) anti-Nrf2 antibody. Relative fold change of K48-linked ubiquitinated Nrf2 was assessed after normalization with respective input lanes; “∗” represents comparison with respect to the MG132-treated mock-infected control.

Apart from the Keap1-Cullin3-Rbx1-dependent canonical Nrf2 turnover pathway, other E3 ubiquitin ligases have been implicated in promoting the proteasomal degradation of Nrf2 [8]. We therefore assessed the status of the Nrf2/HO-1 axis in the presence and absence of a reversible proteasomal inhibitor MG132 under the RV infection scenario. As a positive control for this assay, IRF3, a previously reported substrate for proteasomal degradation during RV infection [17, 85], was simultaneously evaluated. Surprisingly, levels of Nrf2 which were robustly induced in mock-infected MG132-treated cells did not quench following RV-SA11 infection (6 and 9 hpi) (Figures 9(a) and 9(b)). Consistently, levels of HO-1 were also not reduced in RV-SA11-infected cells (6 and 9 hpi) (Figures 9(a) and 9(b)) in the presence of MG132. This suggests the possibility of proteasome-dependent downregulation of the Nrf2/HO-1 axis during RV infection. As expected, RV-mediated IRF3 degradation was also abrogated in the presence of MG132 (Figures 9(a) and 9(b)).

Sensitivity of Nrf2/HO-1 depletion to proteasome inhibition prompted us to investigate possible functional redundancy between Cullin homologs to contribute to Nrf2 regulation during RV-SA11 infection. For this end, depletion of Nrf2 and HO-1 levels was assessed in the presence of the pan-Cullin inhibitor MLN4924. Indeed, levels of both Nrf2 and HO-1 were found to be induced in the presence of MLN4924 under the condition of mock infection (Figure 9(c)). In RV-SA11-infected cells, however, reduction of Nrf2 and HO-1 protein levels was evident distinctly even under the condition of pan-Cullin inhibition (Figure 9(c)). Similar results were obtained even when accumulation of Nrf2 prior to infection was achieved by pretreatment of MLN4924 (Supplementary [Supplementary-material supplementary-material-1]). Disappearance of the neddylated form of Cullin 3 indicated functional inactivation of Cullin following MLN4924 treatment (Figure 9(c)). Insensitivity of RV-SA11-mediated Nrf2 depletion to MLN4924 treatment was further affirmed by confocal microscopy (Figures 9(d) and 9(e)). As observed previously, RV infection was found to cause significant nuclear hollowing of Nrf2 even in MLN4924-treated cells (3.42-fold increase of NH_Q_ value) (Figure 9(f)).

We further assessed the K48-linked ubiquitination status of Nrf2 during infection. Interestingly, after normalization to respective input levels, the reciprocal coimmunoprecipitation assay showed that K48-linked ubiquitinated Nrf2 increased at 9 hpi compared to the mock-infected control (Figures 9(g) and 9(h)). At 3 hpi, a reduction of the K48-linked ubiquitinated Nrf2 was observed (Figures 9(g) and 9(h)). This is indeed justified as redox-regulated induction of Nrf2 includes escape of this transcription factor from the Keap1/Cul3/Rbx1-mediated ubiquitination cycle. The increase of the K48-linked ubiquitination status of Nrf2 at later hours of RV-SA11 infection (9 hpi) becomes more prominent upon inhibition of Cullin3, the E3 ubiquitin ligase responsible for canonical Nrf2 turnover, by MLN4924 (Figures 9(g) and 9(h)). We also visualized K48-linked ubiquitinated Nrf2, which dropped distinctly in response to pan-Cullin inhibition, to get close to the mock-infected control during infection (9 hpi) when we normalized the amount of cellular lysates before immunoprecipitation in accordance with prior normalization of the input lysates such that the levels of Nrf2 remain the same irrespective of treatments (Figures 9(i) and 9(j)). Moreover, with respect to ubiquitylated Nrf2 in mock-infected control, normalized immunoprecipitates also enabled visualization of decreased K48-linked ubiquitination of Nrf2 at 3 hpi and subsequent enrichment of the ubiquitinated Nrf2 stretch at 9 hpi (Figures 9(i) and 9(j)). Proteasomal inhibition also provides another experimental strategy to prevent ubiquitinated pool of Nrf2 to get depleted, enabling assessment of differential Nrf2 ubiquitination in response to different treatments possible by the coimmunoprecipitation assay. In agreement with our hypothesis, we found increased K48-linked ubiquitination of Nrf2 in MG132-treated RV-SA11-infected cells than in MG132-treated mock-infected control (Figures 9(k) and 9(l)).

Recent reports have shown a link between macroautophagy and Nrf2 signaling where autophagy adaptor protein p62 was found to sequester Keap1 away from Nrf2 and to channel it into autophagic flux thereby causing Nrf2 induction [86–88]. Thus, we assessed the contribution of autophagy for modulation of the Nrf2/HO-1 protein expression in RV-SA11-infected cells (9 hpi). Results showed that autophagy inhibitor Bafilomycin A1 could not reverse depletion of Nrf2/HO-1 levels in RV-infected cells (Supplementary [Supplementary-material supplementary-material-1]). Accumulation of lipidated LC3 (LC3-II) in mock- as well as RV-infected cells upon exposure to Bafilomycin A1 confirmed effective inhibition of autophagic flux (Supplementary [Supplementary-material supplementary-material-1]). As was reported previously, LC3-II increase was also prominent in Bafilomycin A1-untreated RV-SA11-infected cells (Supplementary [Supplementary-material supplementary-material-1]) [21, 89]. To our surprise, however, we did not find induced levels of LC3-II in RV-SA11-infected cells to further increase upon Bafilomycin A1 cotreatment (Supplementary [Supplementary-material supplementary-material-1]). This could possibly be due to the redundant mode of action of Bafilomycin A1 and RV infection on autophagolysosome inhibition [89]. As was documented previously [66], Brusatol-mediated Nrf2 depletion was not rescued in the presence of inhibitors against proteasome, CRLs, and autophagy (Supplementary [Supplementary-material supplementary-material-1], [Supplementary-material supplementary-material-1]). In spite of insensitivity of Brusatol-mediated Nrf2/HO-1 depletion to proteasome inhibition, Brusatol has been reported to trigger increased K48-linked ubiquitination of Nrf2 [65]. In our study too, Brusatol treatment was found to exhibit pronounced K48-linked ubiquitinated Nrf2 (Supplementary [Supplementary-material supplementary-material-1], [Supplementary-material supplementary-material-1], [Supplementary-material supplementary-material-1], [Supplementary-material supplementary-material-1]). Moreover, as observed during infection, the increase of K48-linked ubiquitinated Nrf2 in Brusatol-treated cells was more prominent under the condition of the pan-Cullin inhibition (Supplementary [Supplementary-material supplementary-material-1], [Supplementary-material supplementary-material-1], [Supplementary-material supplementary-material-1], [Supplementary-material supplementary-material-1]). Cumulatively, these results suggest that aggravated K48-linked ubiquitination and subsequent proteasomal degradation might result in depletion of the Nrf2/HO-1 axis observed beyond 3 hours of RV-SA11 infection.

## 4. Discussion

Cellular defense feedback elicited in response to viral infection stress essentially serves the purpose of innate antiviral immunity which viruses must overcome to ensure progression of infection. The dynamic interplay between host redox defense response and viral infection associated oxidative stress has multiple regulatory aspects. A modest induction of oxidative insult has been reported to assist viral life cycle and also to contribute to viral pathogenesis in general [90–92]. Citable examples of virus-induced oxidative stress have been documented during infection with human immunodeficiency virus (HIV) [93], influenza A virus (IAV) [94], hepatitis B virus (HBV) [95], hepatitis C virus (HCV) [96], herpes simplex virus (HSV) [97, 98], encephalomyocarditis virus (EMCV) [99], respiratory syncytial virus (RSV) [100], dengue virus (DENV) [101], Japanese encephalitis virus (JEV) [102], and spring viremia of carp virus (SVCV) [103]. When unrestricted, however, exacerbated oxidative menace might potentially trigger apoptotic demise of host cells leading to jeopardized viral perpetuation. Not unsurprisingly, therefore, viral infection has been associated with stabilization of Nrf2 coupled to upregulation of the downstream antioxidant defense to moderate oxidative challenge and to deter abortive apoptotic death of host cells [104–109]. The primary viral trigger behind the mobilized Nrf2/ARE pathway has been shown to be virus-induced oxidative stress as in the case of IAV [107], HIV [106], HSV-1 [110], Kaposi's sarcoma associated herpesvirus (KSHV) [111], DENV [101], SVCV [103], and HBV [112] infections. The importance of ER stress has recently been implicated to result in PERK-dependent activation of the Nrf2/ARE pathway during DENV infection in mononuclear phagocytic cells [113]. Sensitivity of Nrf2/ARE induction upon DENV infection to DPI [101], an antioxidant, as well as GSK2606414 (PERK inhibitor) [113] suggests involvement of both oxidative and ER stress to contribute to host cellular antioxidant boost in either a mutually inclusive or exclusive way. There are also instances of virus-mediated hijacking of cellular proteins which are involved in Nrf2 posttranslational modifications. Classical examples include usurpation of cellular kinases to trigger phosphorylation of Nrf2 leading to redox-independent upsurge of Nrf2/ARE signaling. Combinatorial ectopic expression of DENV NS2B3 has been shown to be responsible for PERK-dependent Nrf2/ARE activation [113]. Implication of immediate early (IE) proteins of human cytomegalovirus (HCMV) has also been evidenced in triggering CKII-mediated Nrf2 activation independent of reactive oxygen species (ROS) [114]. HCV-mediated Nrf2 transactivation, on the other hand, entails involvement of a series of host cellular kinases in a ROS-independent way [115]. Moreover, the essential role of Src, PI3K, and PKC-*ζ* has also been demonstrated in the induction of Nrf2 phosphorylation and activity during infection with KSHV [111]. Elevated Nrf2/ARE signaling during HBV infection has dual causative intermediates—a redox-insensitive pathway via c-Raf/MEK/Erk [105] and another redox-dependent regulation involving ataxia telangiectasia-mutated (ATM)/PKC-*δ*/Nrf2 during HBV-induced carcinogenesis [112]—both of which are triggered by the hepatitis B virus X (HBx) protein. Apart from hijacking the cellular kinase cascade, HBx has also been shown to augment the interaction between Keap1 and p62 thereby liberating Nrf2 from the Keap1-Nrf2 complex, leading to the activation of the Nrf2/ARE pathway [116]. The Marburg virus (MARV) targets Keap1 directly by its protein VP24. Sequestering Keap1 away from Nrf2 is how MARV promotes persistent activation of Nrf2-dependent cytoprotective genes implicated in cellular responses to oxidative stress and regulation of inflammatory reactions [108, 109]. RSV-associated oxidative stress and associated pathophysiology, on the other hand, have been attributed to abrogated activation of the Nrf2/ARE pathway because of proteasomal degradation of Nrf2 by a SUMO-specific E3 ubiquitin ligase RING finger protein 4 (RNF4) [117, 118]. Interestingly, differential influence of viral infection on the Nrf2/ARE pathway is becoming prevalent where context-dependent downregulation of the cellular antioxidant defense has been reported for HBV [119], HCV [120], HIV [121–124], and IAV [125] infection.

In the current study, we observed actively replicating RV to trigger time point-dependent bimodal regulation of Nrf2 in the form of sharp Nrf2 induction from as early as 2 hpi (data not shown) and extending up to 3 hpi followed by gradual decline even below the level of the mock-infected control with increasing time points post infection. Induction accompanied by nuclear enrichment of Nrf2 was further transduced to the transient transcriptional boost followed by a modest protein level accumulation of Nrf2-driven targets such as HO-1 and NQO1 at initial hours of infection (3 hpi). Subsequent studies showed upsurge of Nrf2 to be concurrent with the burst of oxidative stress and highly sensitive to antioxidants but unresponsive to treatments with cellular kinase inhibitors except those against PKC and CKII both of which partially reverted Nrf2 levels. PKC inhibition (by Staurosporine and Gö 6983) also reduced Nrf2 targets in mock-infected as well as in RV-SA11-infected cells, suggesting PKC-driven induction of the Nrf2/HO-1 axis to occur during the initial hours of RV infection, possibly downstream of the RV-induced oxidative burst. K48-linked ubiquitination assay also revealed consistent decrease in relative ubiquitination of Nrf2 at 3 hpi. Beyond the phase of initial induction, depletion of Nrf2 levels with increasing time points of infection (6, 9, and 12 hpi) and subsequent attenuation of Nrf2-driven downstream targets were found to be concurrent with an intensified stretch of polyubiquitinated Nrf2 at 9 hpi. Consistent to the ubiquitin enrichment, proteasomal but not autophagic inhibition prevented Nrf2 depletion post RV infection. Unlike the proteasome, the Keap1/Cul3/Rbx1-based canonical Nrf2 turnover pathway was proven to be dispensable for RV-mediated Nrf2/HO-1 attenuation. Interestingly, K48-linked polyubiquitination of Nrf2 at 9 hpi enriched further in the presence of pan-Cullin inhibitor which prevents canonical Nrf2 turnover. This implies involvement of ubiquitin-proteasome-dependent noncanonical pathway(s) behind depletion of Nrf2 during RV infection. Emerging evidence is accumulating in favour of components of the canonical Nrf2 turnover pathway to contribute to noncanonical regulations of Nrf2. Autophagic modulation of Nrf2 relies on the interaction between Keap1, a critical component of the Nrf2 canonical turnover system, and p62/SQSTM1, a multifunctional adaptor protein for selective autophagy [86–88]. A novel WDR23-DDB1-Cul4 regulatory axis has also been proposed for Nrf2 proteostasis [126]. RV-NSP1 itself has been shown to interact with Keap1, Rbx1, and Cul3 [80, 81]. A previous study on RV-NSP1 interactomics revealed probable association of this RV nonstructural protein with members of CRLs other than Cul3 [81]. Demonstrating persistent Nrf2 depletion after achieving loss-of-function of Keap1, Cul3, and Rbx1 separately and other Cullin homologs as a whole, therefore, nullifies the possibility of noncanonical usurpation of canonical Nrf2 turnover constituents to account for Nrf2 depletion. Surprisingly, the Nrf2/HO-1 axis showed a modest upregulation when RV-NSP1 was overexpressed alone, but this upregulation was overridden when infection was superimposed. RV-NSP1-mediated induction of the Nrf2/HO-1 pathway might possibly arise due to PI3K activation in NSP1 transfected cells [22, 48] or owing to sequestration of the Keap1/Cul3/Rbx1 complex by NSP1 away from Nrf2 [80, 81]. Moreover, Hemin-mediated PI3K-dependent induction of Nrf2 [43] and subsequent Nrf2 target gene expression were also overridden by progressive RV infection. Interestingly, phosphorylation and subsequent inactivation of GSK3*β* downstream of activated PI3K [22] as well as MAP kinase cascade have been shown in RV-infected cells. Activated GSK3*β* is a robust repressor of Nrf2 and exerts its activity at least by two mechanisms. GSK3*β* can directly phosphorylate Nrf2 resulting in its nuclear exclusion and proteasomal degradation via the SCF/*β*-TrCP complex independent of Keap1 [127]. An indirect modulation includes GSK3*β*-mediated activation of Fyn tyrosine kinase which subsequently translocates to the nucleus and phosphorylates Nrf2 at the Tyr568 residue ultimately leading to Nrf2 nuclear exclusion and degradation [128]. In our study, we found RV-SA11 to cause robust nuclear hollowing of Nrf2 under basal as well as nucleus-enriched conditions even in absence of active GSK3*β*.

While drawing a conclusion regarding the probable mechanistic way of Nrf2 depletion in the relatively later hours of RV-infected cells, Nrf2 exhaustion upon Brusatol exposure served as an important positive control. Brusatol, a quassinoid isolated from *Brucea javanica*, an evergreen shrub of Northern Australia and Southeast Asia, has been shown to trigger transient depletion of Nrf2 leading to attenuation of Nrf2-driven transcription units [65, 66]. An exposure with Brusatol for 3 hours curbed basal and Hemin-induced Nrf2/HO-1 independent of the Keap1/Cullin/Rbx1-based canonical Nrf2 turnover pathway [66]. This further authenticated our observation of insensitivity of Nrf2/HO-1 attenuation beyond the initial hours of RV infection to Keap1/Cullin/Rbx1 inhibition. Notably, Brusatol-depleted Nrf2 did not entail Nrf2 nuclear hollowing but was commensurate both in nuclear and in whole cell fractions. Moreover, although having an identical K48-linked polyubiquitination profile of Nrf2 in both Brusatol-treated and RV-infected (9 hpi) cells, RV-depleted Nrf2/HO-1 did restore upon proteasome inhibition whereas Brusatol-drained Nrf2/HO-1 did not [65, 66], suggesting a fundamental difference between the mechanism of these two triggers.

Steady-state Nrf2 protein levels are vulnerable to translational arrest owing to their short half-life span. At least in one report, Nrf2 depletion in Brusatol-exposed cells was shown to be due to global translational inhibition [129]. Rotavirus also triggers severe shut-off of host translation via three mechanisms—preventing translation initiation by PKR-mediated phosphorylation of eIF2*α* [34, 82], RV-NSP3-mediated relocalization of a eukaryotic translational surrogate PABPC1 leading to the abrogated export of nuclear mRNA into cytosol [83], and lastly, ribosomal occupancy of and overhauling by viral messages at the expense of cellular transcripts. Indeed, increasing the life span of Nrf2 upon inhibition of canonical Nrf2 turnover pathway could not block RV-mediated Nrf2 depletion, ruling out the possibility of Nrf2 to get caught in the whirlwind of host translational arrest. Nrf2 translational augmentation even during severe host translational stasis involves regulation of translation from 5′ internal ribosome entry sites (IRES) [9–11]. Emerging evidence suggests augmentative translational element even at the 3′ untranslated region (3′ UTR) of Nrf2 mRNA [12]. Apigenin, a previously reported Nrf2 agonist, has been shown to enhance translation of Nrf2 messages from this 3′ translation regulatory element downstream of the calcium/calmodulin-dependent kinase kinase-*β* (CaMKK*β*)/AMPK signaling [44]. Notably, RV also induced the same signaling downstream of RV-NSP4-induced calcium efflux [21]. However, progressive infection as a whole had an overwhelming effect in the form of overcoming an atmosphere apparently conducive to Nrf2 elevation. PKR inhibition could not restore Nrf2 levels post RV infection. This is indeed justified as RV-mediated attenuation of Nrf2 and HO-1 was shown to be insensitive to treatment with sodium arsenite as well as TM both of which are extremely potent triggers of cellular translational arrest via PERK-mediated eIF2*α* phosphorylation. On the contrary, the Nrf2/ARE pathway is promptly activated upon treatment with a variety of cellular stressors, including the ones causing global translational arrest. Crippling such a primary stress response system along with P body disruption and inhibition of stress granule formation during RV infection would therefore be highly advantageous from a viral perspective [34–38]. In addition, we did not observe rescue of Nrf2/HO-1 depletion following silencing of RV-NSP3. In fact, H_2_O_2_-mediated oxidative Nrf2 stabilization and nuclear translocation were found to be accompanied by cotranslational arrest owing to eIF2*α* phosphorylation and PABPC1 nuclear translocation.

Interestingly, other host proteins with relatively shorter half-lives did not suffer the same fate in RV-infected cells; instead, they are regulated differentially in response to infection. Decrease of p53 during a 2-8-hour window of infection has been reported to be proteasome-sensitive and mediated by RV-NSP1, with the level of p53 restored to that of the control at later hours of infection to orchestrate apoptotic gene induction for ensuring apoptotic dissemination of viral progeny [23]. The trend of p21 follows the same pattern as that of p53—a decline during the span of 2-8 hours followed by restoration. Regulation of p21, however, was found to be transcriptional and not because of global translational arrest [130]. Concurrent with a previous report of overall activation of transcription factor AP1 [46], a sharp upregulation of c-fos was observed at 6 and 9 hours post RV infection. Moreover, Cyclin D1 upregulation during the 2-8-hour window of RV infection was also revealed to be transcriptional and regulated in a calcium/calmodulin/calcium calmodulin-dependent protein kinase I- (CaMKI-) sensitive way [130]. Therefore, host translational shut-off does not impart nonselective hijacking of all cellular messages but rather might be imparted very selectively as to modulate UPR [27] for the ultimate purpose of productive viral replication. Moreover, uncoupling of host translational stasis from stabilization of a short half-life protein Nrf2 and subsequent induction of ARE have been reported in the case of flavivirus infection, further providing evidence for selective manipulation of host stress response machineries by viruses [113, 131]. However, it would be interesting to explore the possibility of whether augmented decoding of Nrf2 mRNA from IRES gets abrogated during RV infection.

Redox stress is prone to rise not only in response to oxidative stressor but also under many other stressful conditions. Therefore, upregulation of the cytoprotective and detoxifying proteins seems to be beneficial not only for disruption of the ROS-dependent steps of viral life cycle but also for amelioration of the exacerbated conditions of infected host cells. In this regard, numerous pharmacological agents were shown to activate the Nrf2 pathway and lessen the burden of virus-induced oxidative stress [132]. The general consensus is that antioxidant therapy either by application of direct cellular antioxidants or through pharmacological upregulation of the cellular antioxidant defense is beneficial for hosts to combat against viral infection. Only in cases of DENV [113] and MARV [109] infection, downregulation of Nrf2/ARE signaling had antagonistic effects on viral replication and pathogenesis. Contrastingly, pharmacological induction of HO-1 by various agonists of the Nrf2/ARE pathway also showed attenuation of DENV infection [133, 134]. We have previously shown RA-839, a highly selective agonist of the Nrf2-ARE pathway, to exert potent antirotaviral efficacy at a subcytotoxic concentration by reducing RV RNA and protein expression, viroplasm formation, and infectious progeny yield *in vitro* [58]. Moreover, CDDO-Me and Hemin, two other Nrf2 inducers, mimicked anti-RV potency of RA-839 [58]. Consistently, antioxidant therapy with NAC has previously been shown to yield remarkable antirotaviral effects [55, 56] and to recuperate clinical patients from rotaviral gastroenteritis [57]. Of significance, antiviral potency of Nrf2 inducers (such as RA-839, tBHQ, CDDO-Me, and Hemin) was found to diminish with the decreasing concentration of inducers and concomitant increase in the load of infectious virions (data not shown). Indeed, we observed Hemin-induced Nrf2 to remain unaffected at low multiplicity of RV-SA11 infection but to get depleted severely with increasing viral dosages. Similar effects of increasing viral load to overwhelm Hemin-induced HO-1 and to override subsequent antiviral effects downstream of HO-1 were observed during infection with Zika virus [135]. This dynamic tussle between host redox stress signaling to restrict viral infection and the viral countermeasure to overwhelm host redox defense provides a fascinating area of host-virus interaction biology in the future.

## 5. Conclusions

**Figure 10:**
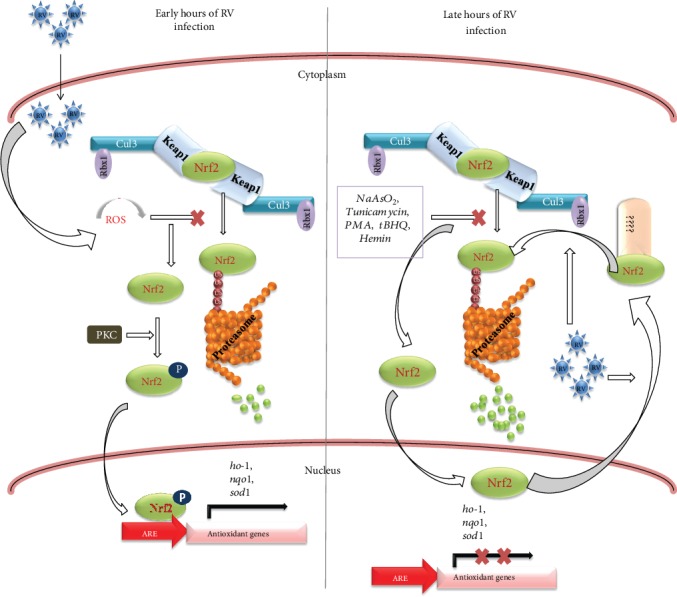
Schematic representation summarizing the modulation of the Nrf2-ARE pathway during RV infection.

Cumulatively, we present here Nrf2, the master regulator of cellular antioxidant defense, to undergo a time point-dependent bimodal regulation in response to RV infection—an initial induction which is dependent on oxidative stress, and partially on PKC, followed by gradual attenuation which is redox-independent but concurrent with increased K48-linked ubiquitination and proteasomal degradation. We have further shown dispensability of the Keap1/Cul3/Rbx1-based canonical Nrf2 turnover pathway and noncanonical Nrf2 regulatory networks involving any of the canonical Nrf2 turnover constituents or even other Cullin(s) with redundant functions in governing downregulation of the Nrf2/HO-1 axis beyond the initial hours of RV infection (Figure 10). With the growing body of evidence on novel Nrf2 repressors such as CR6-interacting factor 1 [136], seven in absentia homolog 2 (SIAH2) [137], RNF4 [118, 138], and synovial apoptosis inhibitor 1 [139] to operate under specific pathophysiological conditions, we are currently in pursuit of identifying the host factors, especially the E3 ubiquitin ligase(s), and also the viral trigger(s) responsible for Nrf2 depletion in RV-infected cells.

## Figures and Tables

**Table 1:** List of reagents used in the study.

Reagents	Catalog no.	Manufacturer
Hemin	H9039	Sigma-Aldrich
Poly(I:C)	P1530	Sigma-Aldrich
Brusatol	SML1868	Sigma-Aldrich
N-Acetylcysteine (NAC)	A7250	Sigma-Aldrich
Ammonium pyrrolidine dithiocarbamate (PDTC)	P8765	Sigma-Aldrich
Diphenyleneiodonium (DPI) chloride	B6326	APExBIO
SB203580	S8307	Sigma-Aldrich
PD98059	M2865-17A.1500	Biomol
SP600125	A4604	APExBIO
LY-294,002 hydrochloride	L9908	Sigma-Aldrich
Staurosporine	S5921	Sigma-Aldrich
DCFDA	D6883	Sigma-Aldrich
GSK2606414	A3448	APExBIO
TBB	A3861	APExBIO
Dorsomorphin	B3252	APExBIO
Tunicamycin (TM)	T7765	Sigma-Aldrich
Sodium arsenite solution	106277	Supelco
PMA	P1585	Sigma-Aldrich
Gö 6983	G1918	Sigma-Aldrich
*tert*-Butylhydroquinone (tBHQ)	112941	Sigma-Aldrich
CDDO-methyl ester	SMB00376	Sigma-Aldrich
RA-839	5707	Tocris
MLN4924	B1036	APExBIO
Bafilomycin A1	19-148	Sigma-Aldrich
MG132	474790	Sigma-Aldrich
Trichostatin A (TSA)	9950	Cell Signaling Technology
C16	527450	Calbiochem
Hydrogen peroxide solution	H1009	Sigma-Aldrich
Protease inhibitor cocktail	P2714	Sigma-Aldrich
Phosphatase inhibitor cocktail	P5726	Sigma-Aldrich
MTT	M5655	Sigma-Aldrich
N-Ethylmaleimide (NEM)	E3876	Sigma-Aldrich

**Table 2:** List of monoclonal and polyclonal antibodies used in the study.

Antibody	Catalog no.	Manufacturer
Nrf2	ab62352	Abcam
HO-1	ab68477	Abcam
HO-2	ab90515	Abcam
NQO1	ab28947	Abcam
GAPDH	sc-25778	Santa Cruz Biotechnology
Acetyl lysine	ab80178	Abcam
pNrf2 (Ser40)	ab76026	Abcam
p-eIF2*α*	9721	Cell Signaling Technology
eIF2*α*	9722	Cell Signaling Technology
Keap1	8047S	Cell Signaling Technology
IRF3	4302S	Cell Signaling Technology
p53	554165	BD Biosciences
FLAG	F1804	Sigma-Aldrich
VP6	sc-101363	Santa Cruz Biotechnology
His	12698	Cell Signaling Technology
Cyclin D1	2978	Cell Signaling Technology
Cul3	sc-136285	Santa Cruz Biotechnology
Rbx1	sc-393640	Santa Cruz Biotechnology
c-fos	2250	Cell Signaling Technology
Anti-VP6	2145	Abcam
p21	2947	Cell Signaling Technology
PABPC1	sc-32318	Santa Cruz Biotechnology
SOD1	2770	Cell Signaling Technology
Histone H3	sc-10809	Santa Cruz Biotechnology
p-PERK	3179	Cell Signaling Technology
PERK	3192	Cell Signaling Technology
K48-linkage-specific Ub	4289	Cell Signaling Technology
LC3-I/II	12741	Cell Signaling Technology

**Table 3:** List of primers used in the study.

Gene name	Primer name	Sequence
*nrf2*	NRF2-forward	5′TGATTCTGACTCCGGCATTT3′
	NRF2-reverse	5′GCCAAGTAGTGTGTCTCCATAG3′

*ho-1*	HO-1-forward	5′ACCAAGTTCAAGCAGCTCTAC3′
	HO-1-reverse	5′GCAGTCTTGGCCTCTTCTATC3′

*ho-2*	HO-2-forward	5′GACCCAGTTCTACCTGTTTGAG3′
	HO-2-reverse	5′CACGATCCTCTCTTTGGTCTTC3′

*nqo1*	NQO1-forward	5′GGGATGAGACACCACTGTATTT3′
	NQO1-reverse	5′TCTCCTCATCCTGTACCTCTTT3′

*sod1*	SOD1-forward	5′GCAGGGCATCATCAATTTCGA3′
	SOD1-reverse	5′TGCAGGCCTTCAGTCAGTCCT3′

*gapdh*	GAPDH-forward	5′GTCAACGGATTTGGTCGTATTG3′
	GAPDH-reverse	5′TGGAAGATGGTGATGGGATTT3′

## Data Availability

The data used to support the findings of this study are available from the corresponding author upon request.

## Supplementary Materials

Supplementary Materials Supplementary Figure 1: (a–d) time point-dependent modulation of Nrf2 protein post infection with different Rotavirus (RV) strains in different RV-permissive cell lines. (e) Status of Nrf2 post transfection with dsRNA analogue Poly(I:C). Supplementary Figure 2: (a) time point-dependent modulation of nrf2, ho-1, ho-2, nqo1 and sod1 mRNA post RV-SA11 infection. (b) Status of basal as well as Hemin-induced HO-1 protein in the presence or absence of Brusatol. Supplementary Figure 3: (a–f) results substantiating initial (3 hpi) induction of Nrf2 post RV infection to be sensitive to antioxidants and partially sensitive to PKC inhibitor. Supplementary Figure 4: status of ROS at 9 hours post mock as well as RV-SA11 infection in the presence and absence of sodium arsenite. Supplementary Figure 5: (a) Brusatol-mediated Nrf2 depletion is not dependent on aggravated Keap1 repression. (b, c) Nrf2/HO-1 depletion post RV infection (9 hpi) remains unperturbed in the presence of reversible and irreversible Keap1 inhibitors. Supplementary Figure 6: Brusatol-mediated Nrf2 depletion is not dependent on Rbx1. Supplementary Figure 7: levels of proteins with relatively short half-lives were shown in a time point-dependent manner post RV-SA11 infection. Supplementary Figure 8: (a, b) inhibition of Cullin homologs and autophagy did not reverse RV-mediated attenuation of the Nrf2/HO-1 axis at 9 hpi. (c, d) Brusatol-mediated Nrf2 depletion is not dependent on Cullin, proteasome, and autophagy. (e–l) Increased K48-linked Nrf2 ubiquitination upon Brusatol treatment.
