# Supplementary material for: Progressive Rotavirus Infection Downregulates Redox-Sensitive Transcription Factor Nrf2 and Nrf2-Driven Transcription Units
Source: Oxid Med Cell Longev. 2020 Apr 4;2020:7289120. doi: 10.1155/2020/7289120 (PMC7165344; doi:10.1155/2020/7289120)
Supplement: Supplementary Materials — Supplementary Figure 1: (a–d) time point-dependent modulation of Nrf2 protein post infection with different Rotavirus (RV) strains in different RV-permissive cell lines. (e) Status of Nrf2 post transfection with dsRNA analogue Poly(I:C). Supplementary Figure 2: (a) time point-dependent modulation of nrf2, ho-1, ho-2, nqo1 and sod1 mRNA post RV-SA11 infection. (b) Status of basal as well as Hemin-induced HO-1 protein in the presence or absence of Brusatol. Supplementary Figure 3: (a–f) results substantiating initial (3 hpi) induction of Nrf2 post RV infection to be sensitive to antioxidants and partially sensitive to PKC inhibitor. Supplementary Figure 4: status of ROS at 9 hours post mock as well as RV-SA11 infection in the presence and absence of sodium arsenite. Supplementary Figure 5: (a) Brusatol-mediated Nrf2 depletion is not dependent on aggravated Keap1 repression. (b, c) Nrf2/HO-1 depletion post RV infection (9 hpi) remains unperturbed in the presence of reversible and irreversible Keap1 inhibitors. Supplementary Figure 6: Brusatol-mediated Nrf2 depletion is not dependent on Rbx1. Supplementary Figure 7: levels of proteins with relatively short half-lives were shown in a time point-dependent manner post RV-SA11 infection. Supplementary Figure 8: (a, b) inhibition of Cullin homologs and autophagy did not reverse RV-mediated attenuation of the Nrf2/HO-1 axis at 9 hpi. (c, d) Brusatol-mediated Nrf2 depletion is not dependent on Cullin, proteasome, and autophagy. (e–l) Increased K48-linked Nrf2 ubiquitination upon Brusatol treatment. [file 7289120.f1.pdf]

(a)

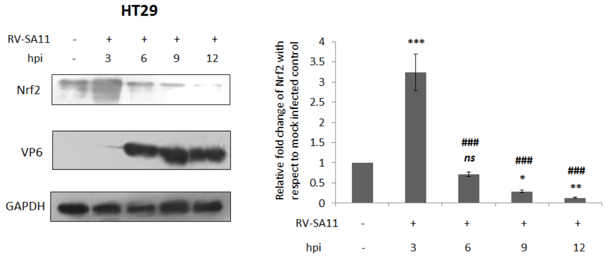

(b)

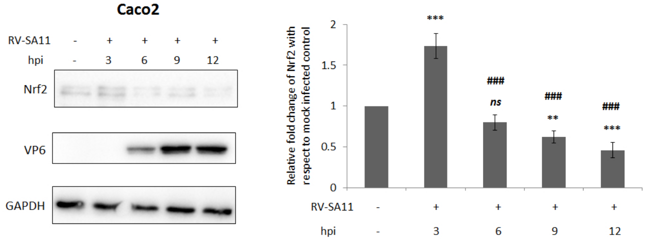

(c)

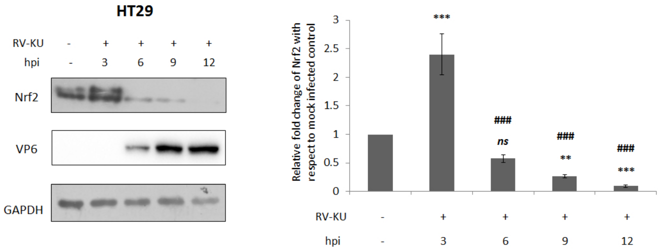

Supplementary Figure 1

(d)

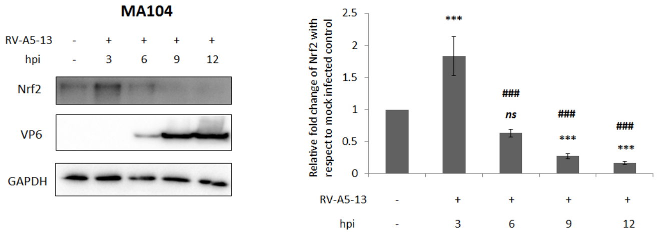

(e)

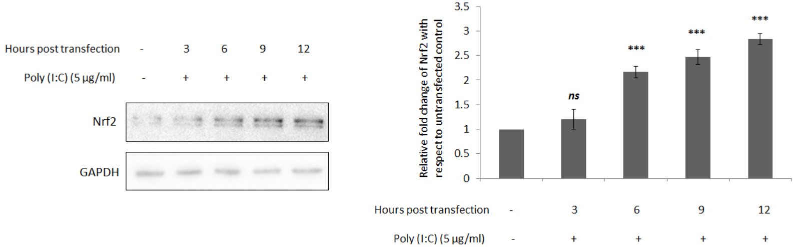

Supplementary Figure 1

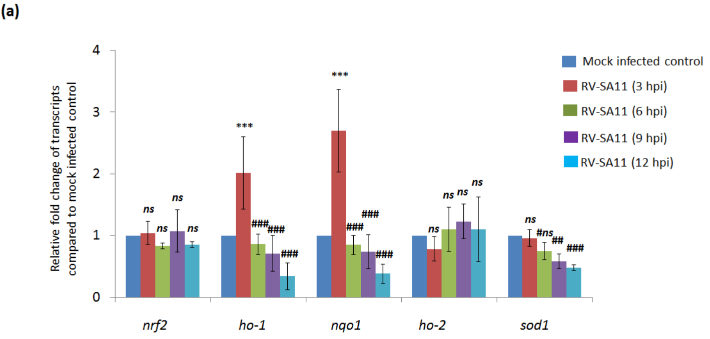

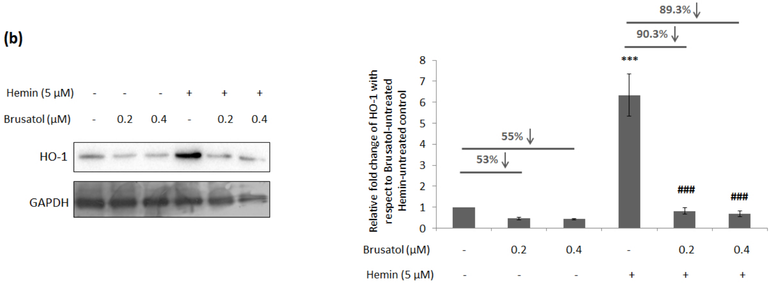

Supplementary Figure 2

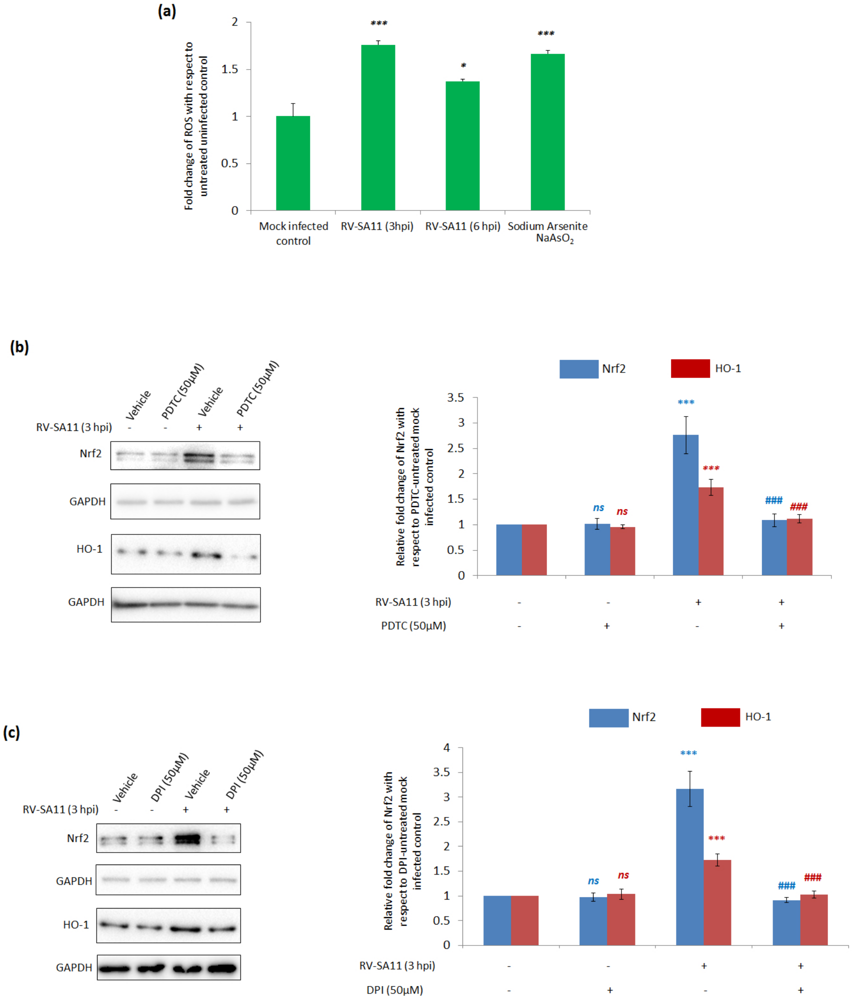

Supplementary Figure 3

(d)

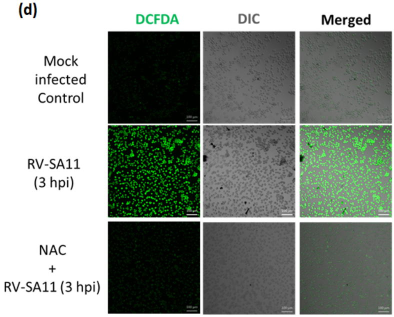

(e)

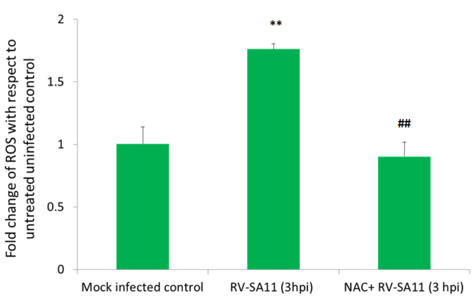

(f)

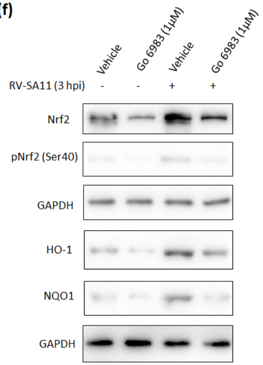

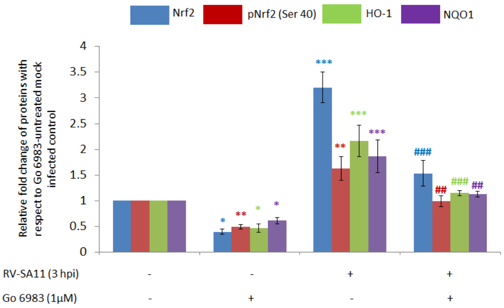

Supplementary Figure 3

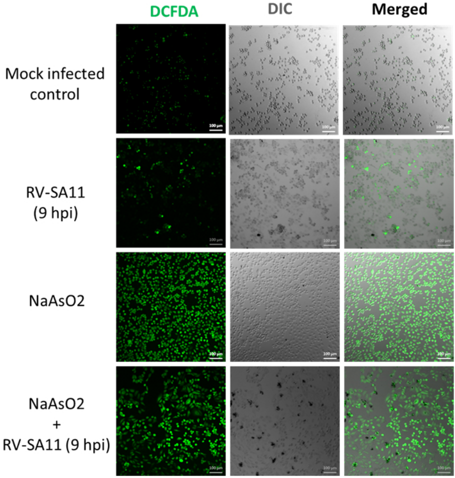

Supplementary Figure 4

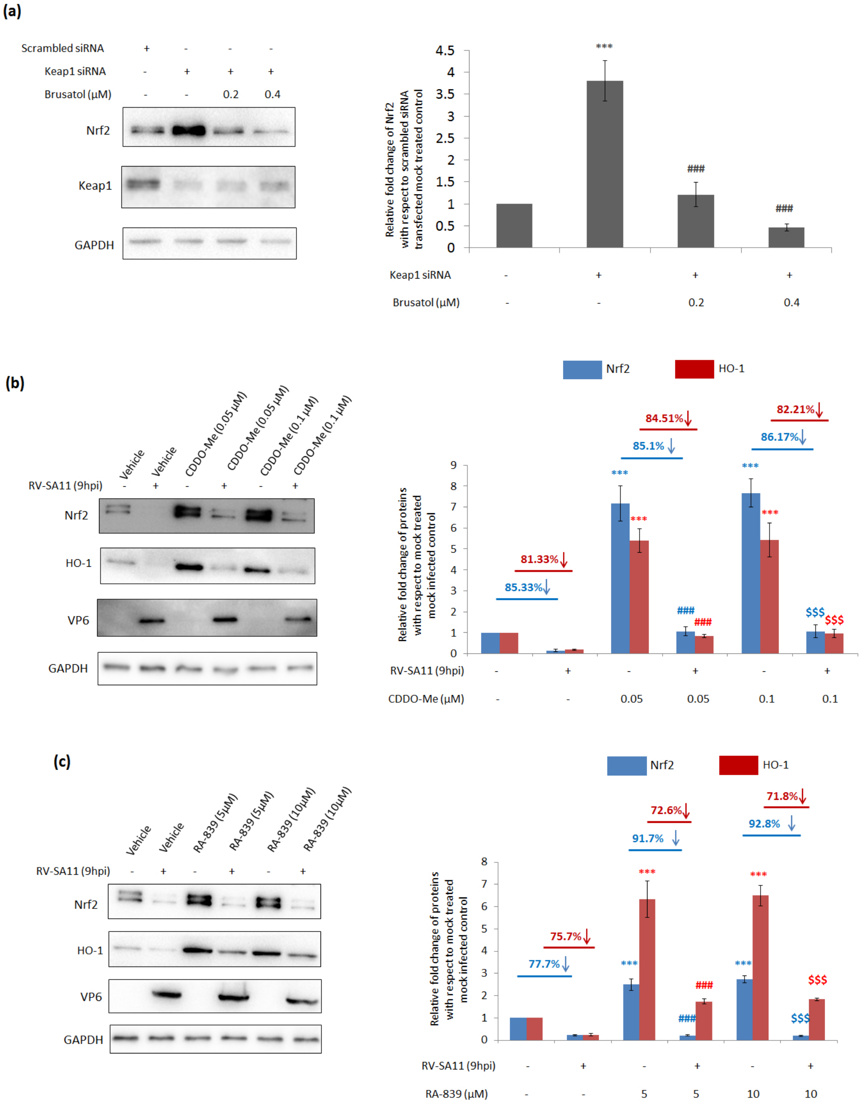

Supplementary Figure 5

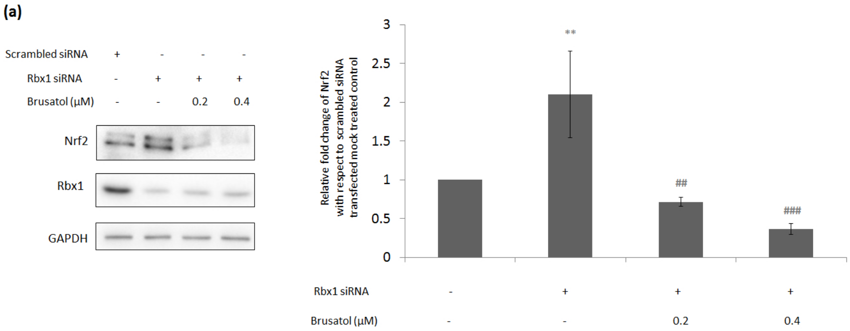

Supplementary Figure 6

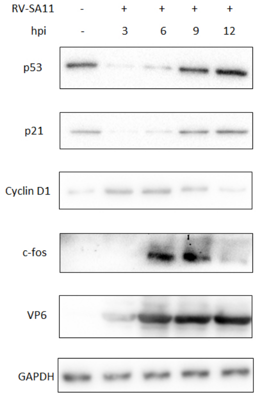

Supplementary Figure 7

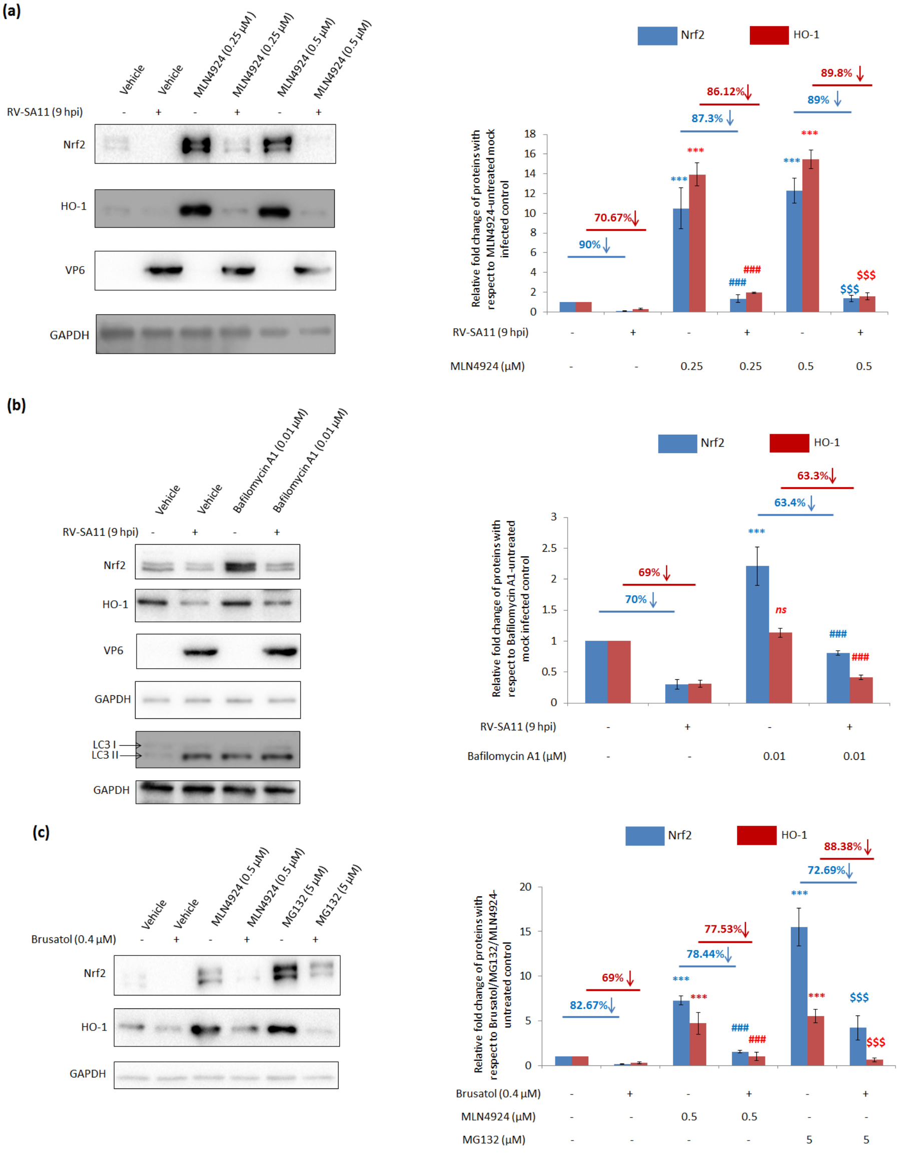

Supplementary Figure 8

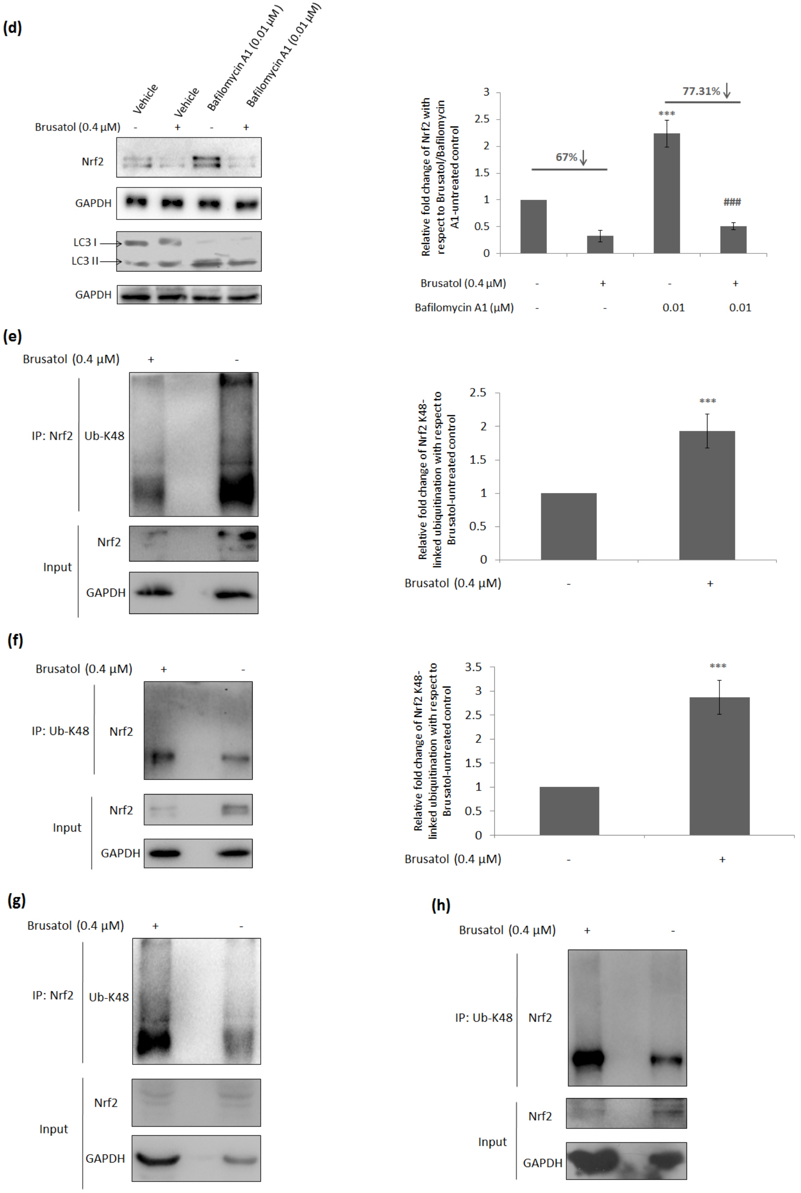

Supplementary Figure 8

(i)

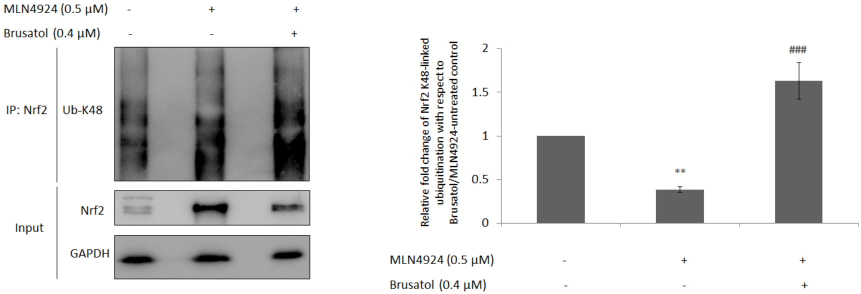

(j)

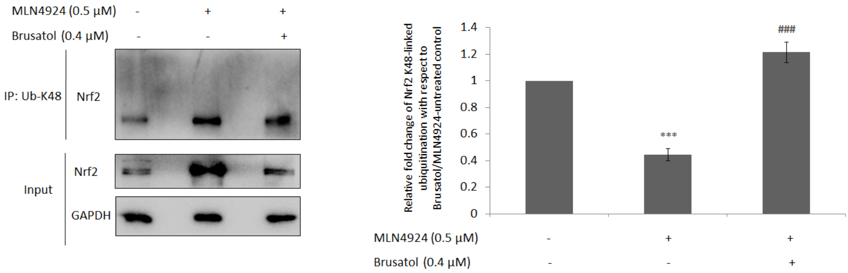

(k)

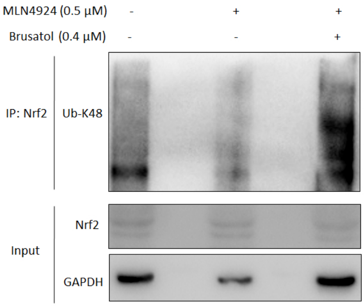

(l)

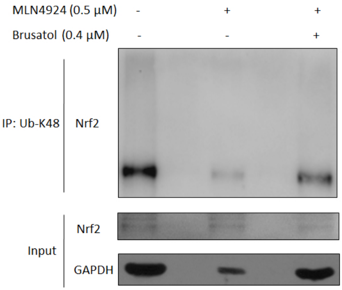

Supplementary Figure 8

Supplementary Figure 1. **(a)** HT29 and **(b)** Caco2 cells were either mock infected or infected with **(a, b)** RV-SA11 for indicated time points. Similarly, **(c)** HT29 and **(d)** MA104 cells were mock-infected/infected with **(c)** RV-KU and **(d)** RV-A5-13, respectively for indicated time points. **(a, b, c, d)** Protein levels of Nrf2 were evaluated in mock and RV infected samples by SDS-PAGE/immunoblot analyses. Relative fold change of Nrf2 is represented; '*ns'* and '*' represent comparisons with respect to mock infected control; '#' represents comparison with respect to RV infected (3hpi) group. (**e**) Nrf2 protein levels were checked in lysates from MA104 cells transfected with Poly(I:C) (5 µg/ml) for 3, 6, 9 and 12 hours by SDS-PAGE followed by immunoblotting. Relative fold change of Nrf2 is represented; '*ns'* and '*' represent comparisons with respect to mock transfected control.

Supplementary Figure 2. (**a**) Total RNA isolated from mock infected and RV-SA11 infected MA104 cells was reverse transcribed and used as templates for qRT-PCR for checking expressions of *nrf2*, *ho-1, ho-2, nqo1* and *sod1*. *gapdh* expression was used as the normalizing control. Relative fold change of gene expression was represented; '*ns'* and '*' represent comparisons with respect to mock infected control; '#*ns*' and '#' represent comparisons with respect to RV infected (3 hpi) group. (**b**) Whole cell extracts of vehicle (H_2O)/Hemin (5 µM) treated MA104 cells further treated with indicated concentration of Brusatol for 3 hours were resolved on SDS-PAGE and HO-1 protein levels were checked by immunoblotting. Relative fold change of HO-1 is represented; '*' and '#' represent comparisons with respect to vehicle treated and Brusatol-untreated Hemin-treated groups, respectively.

Supplementary Figure 3: **(a)** MA104 cells mock infected or infected with RV-SA11 for 3 and 6 hours were further subjected to DCFDA spectrofluorometric assay for measurement of ROS production. A separate group of cells was treated with a well characterized oxidative stressor sodium arsenite ($NaAsO_2$; 50 µM) for 2 hours before measurement of ROS by DCFDA-based spectrofluorometric assay; '*' represents comparison with respect to mock infected control. (**b, c, f**) MA104 cells were mock infected or infected with RV-SA11 for 3 hours in presence or absence of **(b)** PDTC (50 µM; added during final media addition), **(c)** DPI (50 µM; added during final media addition) and **(f)** Gö 6983 (1 µM; added during final media addition). Nrf2, pNrf2 (Ser40), HO-1, NQO1 protein levels were subsequently checked in whole cell extracts by SDS-PAGE/immunoblot analyses. Relative fold changes of proteins are represented. '*ns'* and '*'

represent comparisons with respect to vehicle-treated mock infected control; ‘#’ represents comparison with respect to vehicle-treated RV-SA11 infected (3 hpi) group. (**d, e**) MA104 cells were infected with RV-SA11 for 3 hours in presence or absence of NAC (5 mM) before ROS measurement by DCFDA-based (**d**) confocal imaging and **(e)** spectrofluorometric assay; ‘*’ and ‘#’ represent comparisons with respect to NAC-untreated mock infected and NAC-untreated RV-SA11 infected (3 hpi) groups, respectively.

Supplementary Figure 4: Mock infected and RV-SA11 infected MA104 cells were treated with sodium arsenite ($NaAsO_2$; 50 µM) or vehicle control (H_2O) for 2 hours before processing at 9 hpi for DCFDA-based confocal imaging.

Supplementary Figure 5: (**a**) MA104 cells were transfected with scrambled siRNA/Keap1 siRNA for 36 hours before given exposure to vehicle (DMSO)/Brusatol (0.2 µM and 0.4 µM) for additional 3 hours. Cellular lysates were subsequently run on SDS-PAGE, transferred on to PVDF membrane and probed with anti-Nrf2 as well as anti-Keap1 antibody. Relative fold changes of Nrf2 is represented; ‘*’ and ‘#’ represent comparisons with respect to scrambled siRNA transfected mock treated and Keap1 siRNA transfected mock treated groups, respectively. (**b, c**) MA104 cells were mock infected or infected with RV-SA11 for 9 hours in presence or absence of **(b)** CDDO-Me (0.05 µM and 0.1 µM) (added during final media addition) and **(c)** RA-839 (5 µM and 10 µM) (added during final media addition). **(b, c)** Nrf2 and HO-1 protein levels were subsequently checked in whole cell extracts by SDS-PAGE/immunoblot analyses. Relative fold changes of proteins are represented; for CDDO-Me treatment groups, ‘*’, ‘#’ and ‘$’ represent comparisons with respect to vehicle treated mock infected, CDDO-Me (0.05 µM)-treated mock infected and CDDO-Me (0.1 µM)-treated mock infected groups, respectively. For RA-839 treatment groups, ‘*’, ‘#’ and ‘$’ represent comparisons with respect to vehicle treated mock infected, RA-839 (5 µM)-treated mock infected and RA-839 (10 µM)-treated mock infected groups, respectively.

Supplementary Figure 6: **(a)** Rbx1 siRNA transfected MA104 cells (kept for 36 hours) were treated with Brusatol (0.2 µM and 0.4 µM) or vehicle control (DMSO) for 3 hours. Nrf2 and Rbx1 levels were subsequently assessed in cellular extracts by SDS-PAGE/immunoblot analyses. Relative fold change of Nrf2 is represented; ‘*’ and ‘#’ represent comparisons with

respect to scrambled siRNA transfected Brusatol-untreated and Rbx1 siRNA transfected Brusatol-untreated groups, respectively.

Supplementary Figure 7: Protein levels of p53, p21, c-fos, cyclin D1 were checked in MA104 cells at indicated time point post RV-SA11 infection.

Supplementary Figure 8: **(a)** MA104 cells pre-treated with MLN4924 (0.25 µM and 0.5 µM) for 6 hours were mock infected or infected with RV-SA11. Nrf2 and HO-1 protein levels were assessed in cellular extracts prepared 9 hours after infection by SDS-PAGE/immunoblot analyses. Relative fold changes of proteins are represented; '*', '#' and '$' represent comparisons with respect to vehicle-treated mock infected, MLN4924 (0.25 µM)-treated mock infected and MLN4924 (0.5 µM)-treated mock infected groups, respectively. **(b)** Mock infected and RV-SA11 infected MA104 cells were treated with Bafilomycin A1 (0.01 µM) or vehicle control (DMSO) during final media addition (1 hpi). Protein levels of Nrf2, HO-1 and LC3-I/II were assessed in cellular extracts prepared at 9 hpi by immunoblot analyses. Relative fold changes of proteins are represented; '*ns*' and '*' represent comparisons with respect to vehicle-treated mock infected control; '#' represents comparison with respect to Bafilomycin A1-treated mock infected group. **(c)** Vehicle control (DMSO)/MLN4924 (0.5 µM)/MG132 (5 µM) treated MA104 cells (for 3 hours) were co-treated with Brusatol (0.4 µM) or kept Brusatol-untreated for an additional 3 hours. Cellular lysates were subjected to SDS-PAGE/immunoblot analyses for checking protein levels of Nrf2 and HO-1. Relative fold changes of proteins are represented; '*', '#' and '$' represent comparisons with respect to vehicle-treated Brusatol-untreated, MLN4924 (0.5 µM)-treated Brusatol-untreated and MG132 (5 µM)-treated Brusatol-untreated groups, respectively. (**d**) Bafilomycin A1 (0.01 µM) pre-treated MA104 cells were co-treated with Brusatol (0.4 µM) for an additional 3 hours before cellular lysates were prepared and subjected to SDS-PAGE/immunoblotting for checking Nrf2 and LC3-I/II expression. Relative fold change of Nrf2 is represented; '*' and '#' represent comparisons with respect to vehicle-treated Brusatol-untreated and Bafilomycin A1-treated Brusatol-untreated groups, respectively. (**e, f**) Lysates from DMSO/Brusatol (0.4 µM)-treated cells (exposure of 3 hours) were subjected to immunoprecipitation with (**e**) anti-Nrf2 or (**f**) anti-K48-Ub antibody. Immunoprecipitates were subsequently run on SDS-PAGE and probed with (**e**) anti-K48-linked Ub or (**f**) anti-Nrf2 antibody. Presence of Nrf2 was evaluated in input lysates. Relative fold change of K48-linked

ubiquitinated Nrf2 was assessed after normalization with respective input lanes; '*' represents comparison with respect to Brusatol-untreated control. (**g, h**) Lysates from DMSO/Brusatol (0.4 µM)-treated cells (exposure of 3 hours) were subjected to immunoprecipitation with (**g**) anti-Nrf2 or (**h**) anti-K48-Ub antibody. Amount of cellular lysates which were subjected to immunoprecipitation (to assess K48-linked ubiquitinated Nrf2) were normalized on the basis of prior normalization of Nrf2 input levels such that Nrf2 levels remain the same in each input lane. Immunoprecipitates were subsequently run on SDS-PAGE and probed with (**g**) anti-K48-linked Ub or (**h**) anti-Nrf2 antibody. Presence of Nrf2 was evaluated in input lysates. (**i, j**) Lysates from DMSO/MLN4924 (0.5 µM)-treated (exposure of 3 hours) MA104 cells co-treated with DMSO/Brusatol (0.4 µM) (additional exposure of 3 hours) were subjected to immunoprecipitation with (**i**) anti-Nrf2 or (**j**) anti-K48-Ub antibody. Immunoprecipitates were subsequently run on SDS-PAGE and probed with (**i**) anti-K48-linked Ub or (**j**) anti-Nrf2 antibody. Presence of Nrf2 was evaluated in input lysates. Relative fold change of K48-linked ubiquitinated Nrf2 was assessed after normalization with respective input lanes; '*' and '#' represent comparisons with respect to Brusatol-untreated MLN4924-untreated and Brusatol-untreated MLN4924-treated groups, respectively. (**k, l**) Lysates from DMSO/MLN4924 (0.5 µM)-treated (exposure of 3 hours) MA104 cells co-treated with DMSO/Brusatol (0.4 µM) (additional exposure of 3 hours) were subjected to immunoprecipitation with (**k**) anti-Nrf2 or (**l**) anti-K48-Ub antibody. Amount of cellular lysates which were subjected to immunoprecipitation (to assess K48-linked ubiquitinated Nrf2) were normalized on the basis of prior normalization of Nrf2 input levels such that Nrf2 levels remain the same in each input lane. Immunoprecipitates were subsequently run on SDS-PAGE and probed with (**k**) anti-K48-linked Ub or (**l**) anti-Nrf2 antibody. Presence of Nrf2 was evaluated in input lysates.
